# Expression of Mucin Family Proteins in Non-Small-Cell Lung Cancer and its Role in Evaluation of Prognosis

**DOI:** 10.1155/2022/4181658

**Published:** 2022-08-26

**Authors:** Jing Tu, Min Tang, Guoqing Li, Liang Chen, Yubo Wang, Yong Huang

**Affiliations:** ^1^Department of Pulmonary and Critical Care Medicine, Chongqing General Hospital, No. 118, Xingguang Avenue, Liangjiang New Area, Chongqing 401147, China; ^2^Department of Oncology, Chongqing General Hospital, No. 118, Xingguang Avenue, Liangjiang New Area, Chongqing 401147, China; ^3^Intensive Care Unit, Chongqing General Hospital, No. 118, Xingguang Avenue, Liangjiang New Area, Chongqing 401147, China; ^4^Department of Respiratory Medicine, Daping Hospital, Army Medical University, Chongqing 400042, China

## Abstract

Lung cancer is still the major contributor to cancer-related mortality. Over 85% of patients suffer from non-small-cell lung cancer (NSCLC). Mucins (MUCs) are large glycoproteins secreted or membrane-bound produced by epithelial cells in normal and malignant tissues. They are the major components of the mucous gel that covers the surface of the respiratory epithelium. Certain MUCs have been used or proposed to act as biomarkers for lung cancer. Nevertheless, the expression, messenger ribonucleic acid (mRNA) levels, and the prognostic value of MUCs in NSCLC are yet to be investigated systematically. In this research, the biological information of MUC proteins in patients with NSCLC was examined using a series of databases. The results based on gene expression profiling interactive analysis (GEPIA) illustrated that the expression *of MUC3A, MUC4, MUC5B, MUC13, MUC16,* and *MUC21* mRNAs was remarkably upmodulated in lung adenocarcinoma (LUAD) patients, whereas the *MUC1* expression was downregulated in lung squamous cell carcinoma (LUSC) patients. Kaplan–Meier plotter (KM Plotter) analysis revealed that elevated mRNA expression levels of *MUC3A* and *MUC16* were linked to unfavourable overall survival (OS) in NSCLC, while increased mRNA expression of *MUC1* and *MUC15* was linked to good OS, especially in LUAD patients. In addition, differential expression of *MUC1*, *MUC3A/3B*, *MUC8*, *MUC12*, *MUC15*, and *MUC16* mRNA was linked to the prognoses of NSCLC patients with varied clinical-pathological subtypes. Genetic alterations of MUCs in NSCLC primarily involved mutations, fusion, amplification, deep deletion, and multiple alterations according to cancer genomics (cBioPortal). Therefore, we propose that combinations of MUC proteins can act as prognostic biomarkers and demonstrate the therapeutic potential for NSCLC-related therapy.

## 1. Introduction

Lung cancer continues to be one of the world's fatal cancers. The most frequently diagnosed histological subtype of lung cancer is non-small-cell lung cancer (NSCLC) which is responsible for over 85 percent of all lung cancer cases. NSCLC has two main histological phenotypes namely, lung adenocarcinoma (LUAD, attributed to around 50% of all cases) and lung squamous cell carcinoma (LUSC, attributed to around 40% of all cases) [1]. A majority of patients having early-stage lung cancer are typically asymptomatic or demonstrate distant metastasis at the first diagnosis. People who are diagnosed with metastatic NSCLC had a 5-year overall survival chance of lower than 5% in the previous decade [2]. Although the tumour-node-metastasis (TNM) staging system helps to decide suitable strategies for NSCLC treatment, the survival rates among NSCLC patients who are at the same stage and receiving the same therapy might vary remarkably [1]. Hence, it is crucial to explore effective tumour biomarkers for assisting early diagnosis, prognosis evaluation, and appropriate treatment for NSCLC.

Mucins (MUCs) are a group of glycoconjugates with high molecular weight for protecting epithelial cells as a physical barrier. However, recent research proves that they are involved in tumour development, tumour cell growth, and immune escape by altering localization or glycosylation patterns [3]. To date, there are 21 MUC genes in humans that have been discovered and confirmed by the HUGO Gene Nomenclature Committee (HGNC). Some MUC genes have already been demonstrated to have prognostic values in different cancer types. For instance, MUC16 (CA125) is a well-known cancer biomarker contributing to disease progression and metastasis in several malignancies [4, 5]. MUC12 was identified as a candidate gene involved in colorectal cancer (CRC) metastasis and was an independent prognostic factor in stages II and III CRC [6]. The elevated expression level of MUC15 was linked to survival in stomach adenocarcinoma [7]. MUC13 is commonly dysregulated in diverse epithelial carcinomas, including gastric, colorectal, and ovarian malignancies [8]. Jonckheere et al. discovered an MUC4/MUC16/MUC20 signature that was associated with poor survival in pancreatic, colon, and stomach cancers [9]. MUC21 was considered a potential biomarker for assisting LUAD diagnosis and treatment [10].

**Figure 1:**
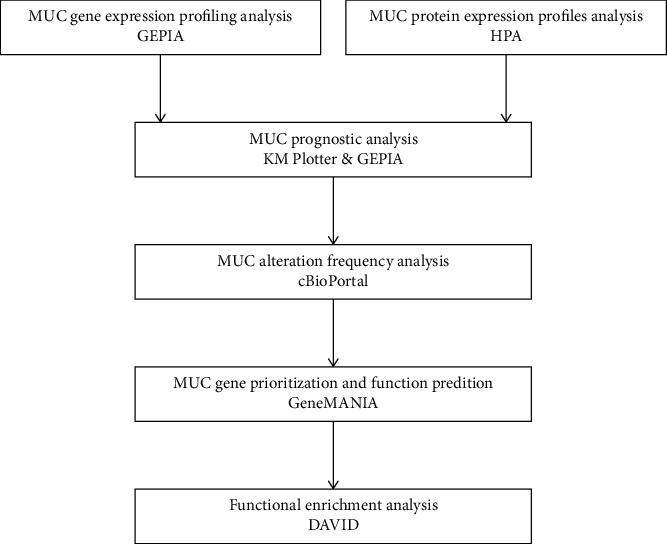
Work flow of databases mining for MUC in this study. MUC, mucins. GEPIA: Gene Expression Profiling Interactive Analysis. HPA: Human Protein Atlas. KM: Kaplan-Meier. DAVID: Database for Annotation, Visualization, and Integrated Discovery.

Nevertheless, the role of expression, prospective functions, and the prognostic significance of MUCs in the prognosis of NSCLC is still contentious and has not been explored systematically. This might be attributed to the complexity of MUC biology and the existence of multiple MUCs with differing functions within different cells at various stages [11]. We hypothesised that combinations of MUC proteins could act as prognostic biomarkers for NSCLC treatment. Considering that human airway located MUCs are possibly involved in the development of NSCLC, this study selected 19 human *MUC* genes (*MUC1*, *MUC2*, *MUC3A*, *MUC3B*, *MUC4*, *MUC5AC*, *MUC5B*, *MUC6*, *MUC7*, *MUC8*, *MUC12*, *MUC13*, *MUC15*, *MUC16*, *MUC17*, *MUC19*, *MUC20*, *MUC21*, and *MUC22*) that are highly abundant in the human airway for bioinformatics analysis based on the expression profiles of LUSC and LUAD patients. Several online tools for data mining were used for investigating the MUC family members' expression, function, and prognostic value in NSCLC (Figure 1).

## 2. Materials and Methods

### 2.1. Analysis of Gene Expression Profiles

NSCLC cohorts with gene expression profiles, gene variation data, and clinical information were used in this study. GEPIA (gene expression profiling interactive analysis,https://gepia.cancer-pku.cn) [12] was employed to analyse the RNA sequence expression profile of NSCLC and adjacent tumour tissues from the Cancer Genome Atlas (TCGA) and Genotype-Tissue Expression (GTEx) project. The MUC expression in tumour and normal specimens was subjected to an analysis utilizing the Student's *t*-test, and the MUC expression in various stages of NSCLC was investigated utilizing the *F*-test. *P* < 0.01; the fold change (FC) >2 was established as the parameters for determining a significant difference. Additionally, MUC protein expression profiles available from the Human Protein Atlas database (HPA) (https://www.proteinatlas.org/) were compared to find out the possible matched expression at the protein and mRNA levels. In the cell types annotated, antibody (Ab) staining levels ranged from nondetected, low, medium to high. The staining degree and proportion of stained cells were utilized to compute the score [13–15].

### 2.2. Prognostic Analysis

The Kaplan–Meier (KM) plotter (https://www.kmplot.com) is a platform available for analyzing the impact of 54 k genes (protein, miRNA, and mRNA) on the survival of 21 distinct kinds of cancer, such as gastric (*n* = 1,440), lung (*n* = 3,452), ovarian (*n* = 2,190), and breast (*n* = 6,234) cancers from Gene Expression Omnibus (GEO), the European Genome-phenome Archive (EGA), and TCGA databases. The KM plotter's primary objective is to undertake a metaanalysis-based identification and verification of survival biological markers [16]. The KM plotter and GEPIA were both employed to assess the predictive significance of MUC mRNA expression. We also analysed the disease-free survival (DFS) and OS of NSCLC patients. Subsequently, the patient specimens were categorized into high- and low-expression groups predicated on median mRNA expression, log-rank *P*-values, and hazard ratios (HR) with 95% confidence intervals (CI) [17, 18]. Statistical significance was established as log-rank *P*-values <0.05. Univariate Cox analysis was undertaken with adjustments to several groups based on different clinicopathological features, namely, sex, chemotherapy, clinical stages, and smoking status among patients with NSCLC.

### 2.3. Analyses of the Frequency of Gene Mutations

MUC gene mutations in patients with NSCLC were examined with visualization and analysis of the following datasets: cBioPortal for cancer genomics (https://www.cbioportal.org) [19, 20]. Genomic profiles were selected by screening individual *MUC* gene symbols for parameters such as cancer studies, levels of mRNA expression, putative copy-number alterations (CNV), and mutations.

### 2.4. Bioinformatics Analysis and Functional Enrichment

For gene-level correlation analysis, GeneMANIA (https://www.genemania.org), a biological network integrative platform for the prioritization of genes and prediction of their functions, was utilized [21]. We conducted gene ontology (GO) terms and Kyoto Encyclopedia of Genes and Genomes (KEGG) pathways enrichment analyses [22, 23] with DAVID version 6.8 (https://david.ncifcrf.gov/tools.jsp).

### 2.5. Statistical Analysis

All statistical analyses were performed during the analysis in online bioinformatics tools. Students' *t*-test was conducted between the two groups. The ANOVA test was conducted among three or over three groups. The log-rank test was conducted in Kaplan–Meier survival analysis. *P* < 0.05 was considered significant.

## 3. Results

### 3.1. Levels of MUC mRNA in Patients with NSCLC

**Figure 2:**
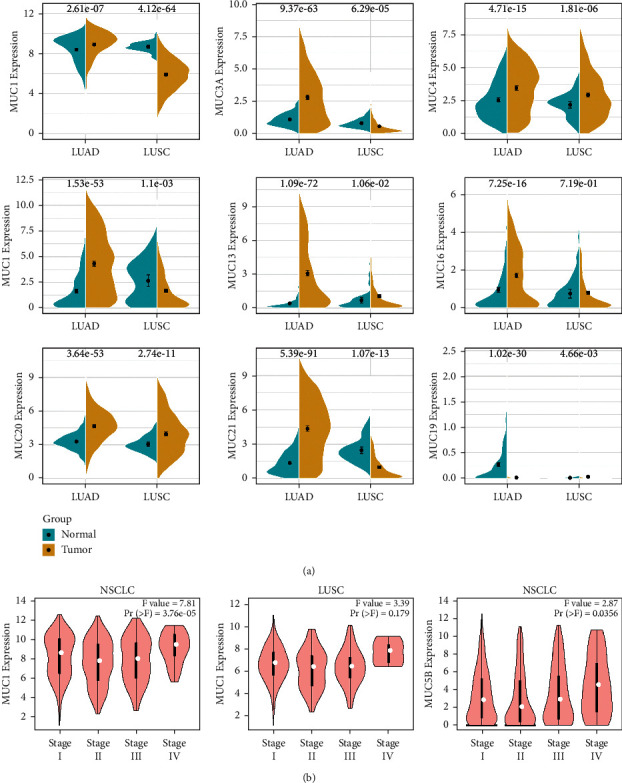
(a) Expression of MUC in LUAD and LUSC compared with that in normal tissues using the GEPIA database. (b) Expression of MUC during different stages of NSCLC in the GEPIA database. The threshold was analysed by the *F*-test, *P*-value = 0.01, and fold change (FC) = 2, data type: mRNA. (T) tumour; (N) normal; MUC: mucins; LUAD: lung adenocarcinoma; LUSC: lung squamous cell carcinoma; mRNA: messenger ribonucleic acid; GEPIA: Gene Expression Profiling Interactive Analysis.

GEPIA was utilized to analyse the relative MUC mRNA expression in LUSC and LUAD as opposed to that in normal tissues. *MUC3A*, *MUC4*, *MUC5B*, *MUC13*, *MUC16*, and *MUC21* mRNA expression levels were considerably elevated in LUAD as opposed to those in normal lung specimens. In addition, the *MUC20* mRNA expression level was remarkably increased in both LUSC and LUAD in contrast with that in normal lung specimens. Contrastingly, the *MUC1* mRNA expression level was considerably reduced in LUSC as opposed to that in normal lung samples (Figure 2(a)).

*MUC* expression was also studied during I, II, III, and IV stages of NSCLC (Figure 2(b)). The findings illustrated that the levels of *MUC1* and *MUC5B* mRNA expression changed considerably across various tumour stages (*P* < 0.05). Especially, the expression level of MUC5B in stage IV was almost twice in stage II. However, the mRNA expression of other *MUC* genes did not differ among tumour stages. *MUC* mRNA expression at different clinical stages was also studied in LUSC and LUAD. The findings illustrated that the mRNA expression level of *MUC1* changed significantly across various tumour stages in LUSC, being higher at stages I and IV than at stages II and III. Furthermore, the levels of *MUC1*, *MUC2*, *MUC3A*, *MUC4*, *MUC5AC*, *MUC5B*, *MUC6*, *MUC7*, *MUC12*, *MUC13*, *MUC15*, *MUC16*, *MUC17*, and *MUC21* mRNA expressions matched the reported protein expression levels in the HPA database. However, the representative images of the protein levels of MUC3B, MUC8, MUC19, MUC20, and MUC22 were unavailable in the HPA database. Immunohistochemistry (IHC) results from the HPA database displayed that MUC1 and MUC5B expressions were strikingly elevated among IHC data of eight available MUC genes in both LUAD and LUSC compared with the normal tissue (Supplementary Figure 1).

### 3.2. Prognostic Significance of MUC mRNA Levels in NSCLC

**Figure 3:**
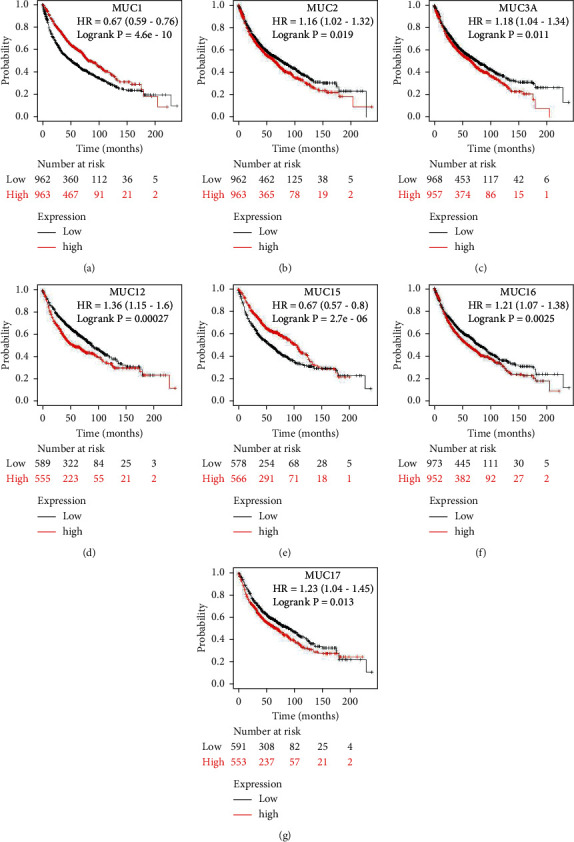
Correlation between MUC mRNA expression and OS in NSCLC analysed using the KM plotter. OS curves of (a) MUC1 (Affymetrix IDs: 213693_s_at), (b) MUC2 (Affymetrix IDs: 204673_at), (c) MUC3A (Affymetrix IDs: 214676_x_at), (d) MUC12 (Affymetrix IDs: 226654_at), (e) MUC15 (Affymetrix IDs: 227238_at), (f) MUC16 (Affymetrix IDs: 220196_at), and (g) MUC17 (Affymetrix IDs: 232321_at). OS survival curves comparing NSCLC patients with high (red) and low (black) MUC expression was plotted, with a threshold *P*-value <0.05. MUC: mucins; OS: overall survival; HR: Hazard Ratio; NSCLC: non-small-cell lung cancer; KM: Kaplan-Meier.

**Figure 4:**
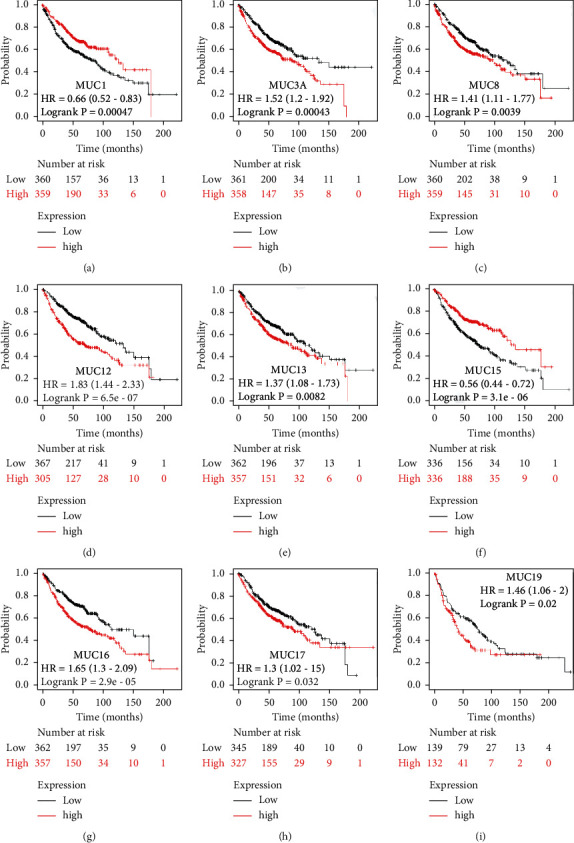
Correlation between MUC mRNA expression and OS in LUAD analysed by the KM Plotter. OS curves of (a) MUC1 (Affymetrix IDs: 213693_s_at), (b) MUC3A (Affymetrix IDs: 214676_x_at), (c) MUC8 (Affymetrix IDs: 217295_at), (d) MUC12 (Affymetrix IDs: 226654_at), (e) MUC13 (Affymetrix IDs: 218687_at), (f) MUC15 (Affymetrix IDs: 227238_at), (g) MUC16 (Affymetrix IDs: 220196_at), and (h) MUC17 (Affymetrix IDs: 232321_at). (i) Correlation between MUC mRNA expression and OS in LUSC analysed using the KM plotter. OS curves of MUC1 9 (Affymetrix IDs: 1553436_at). OS survival curves comparing LUAD patients with high (red) and low (black) MUC expression were plotted, with a threshold *P*-value <0.05. MUC: mucins; OS: overall survival; HR: Hazard Ratio; LUAD: lung adenocarcinoma; KM: Kaplan-Meier.

MUC levels were evaluated for prognostic significance utilizing the KM plotter analysis in both the whole NSCLC cohort and the LUSC and LUAD subtypes. Increased MUC1 and MUC15 mRNA expression levels were linked to a favourable OS in the whole cohort. In contrast, an increase in *MUC2*, *MUC3A*, *MUC12*, *MUC16*, and *MUC17* mRNA expressions was strongly linked to the unfavourable OS in NSCLC (Figures 3(a)–3(g)). In addition, increased *MUC1* and *MUC15* mRNA levels were linked to favourable OS, and elevated *MUC3A*, *MUC8*, *MUC12*, *MUC13*, *MUC16*, and *MUC17* mRNA levels were linked to unfavourable OS among LUAD patients (Figures 4(a)–4(h)). Moreover, elevated mRNA levels of *MUC19* were considerably linked to unsatisfactory OS among patients with LUSC (Figure 4(i)). Notably, these results indicated that *MUC1*, *MUC3A*, *MUC8*, *MUC12*, *MUC13*, *MUC15*, *MUC16*, and *MUC17* perform different prognostic functions in LUAD.

**Figure 5:**
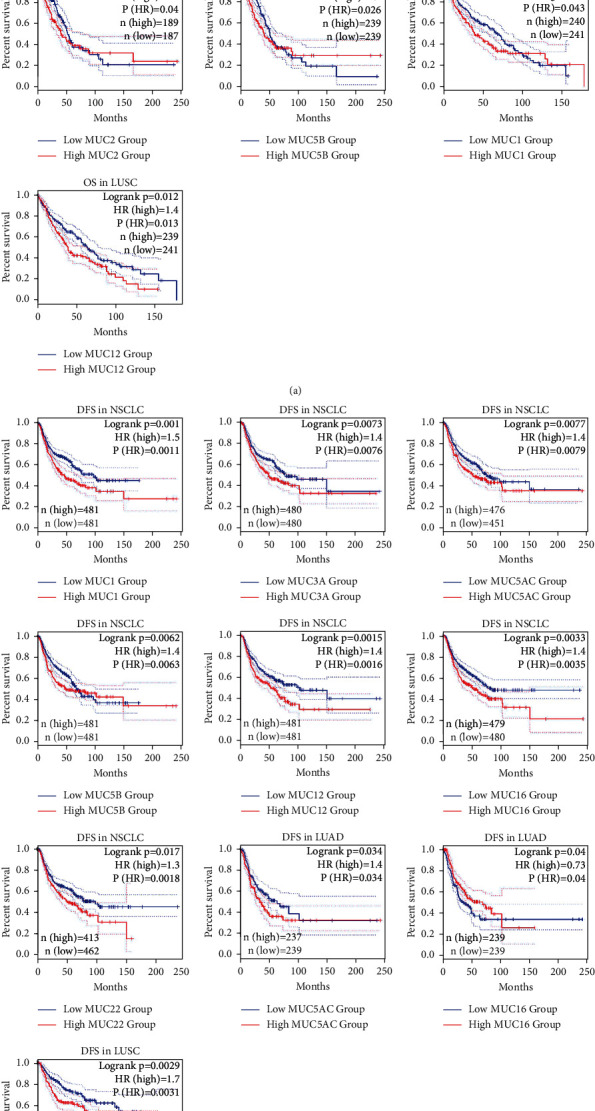
Validation of MUC prognostic values in NSCLC using GEPIA. (a) Correlation between MUC mRNA expression and OS in NSCLC, LUAD, and LUSC. (b) Correlation between MUC mRNA expression and DFS in NSCLC, LUAD, and LUSC. The complete line represents the survival curve, with 95% confidence interval and was marked as a dotted line. Survival curves compared high (red) and low (blue) MUC expression levels in patients with NSCLC. MUC: mucins; GEPIA: Gene Expression Profiling Interactive Analysis; OS: overall survival; DFS, disease free survival; NSCLC: non-small-cell lung cancer; LUAD: lung adenocarcinoma; LUSC: lung squamous cell carcinoma.

Additionally, members of the MUC family were verified utilizing NSCLC data acquired from the GEPIA database. As depicted in Figure 5(a), increased *MUC2*, *MUC12*, and *MUC16* mRNA expression levels were linked to unfavourable OS in NSCLC patients. The expression of *MUC* mRNA levels was subsequently studied in LUAD and LUSC. Increased *MUC2* and *MUC5B* mRNA expressions were linked to the adverse OS in LUAD patients, and increased expressions of *MUC1* and *MUC12* mRNA in LUSC patients were associated with adverse OS. Other MUCs did not demonstrate any correlation with the OS. Increased *MUC1*, *MUC3A*, *MUC5AC*, *MUC5B*, *MUC12*, *MUC16*, and *MUC22* mRNA expressions correlated with unfavourable DFS in NSCLC patients (Figure 5(b)). An increase in *MUC5AC* mRNA expression was linked to adverse DFS in LUAD patients, whereas an increase in *MUC21* mRNA expression was related to good DFS in LUAD patients. Besides, increased *MUC12* mRNA expression correlated with unfavourable DFS in LUSC patients.

### 3.3. MUC mRNA Level Prognostic Significance in NSCLC Subsets with Various Clinical-Pathological Characteristics

The association between *MUC* mRNA level expression and different clinical-pathological features, which include chemotherapy, clinical stages, smoking history, and sex, was evaluated in the NSCLC subsets. *MUC3A* and *MUC3B* were used as the probe in the KM plotter; whereas, *MUC21* and *MUC22* were not available on the platform. It was observed that a high *MUC15* mRNA level was linked to favourable OS in patients with a smoking history in LUAD. In contrast, high *MUC12* and *MUC16* mRNA levels were linked to unfavourable OS among patients with a smoking history in LUAD. High *MUC8* mRNA levels were related to unfavourable OS in patients with smoking in LUSC. Whereas, high *MUC15* mRNA levels were linked to favourable OS in smokers with LUAD (Supplementary Tables 1A–1C). *MUC3A/3B*, *MUC5B*, *MUC8*, *MUC12*, and *MUC13* mRNA expressions had a considerable link to unfavourable OS in patients with early-stage LUAD. However, *MUC15* and *MUC19* were linked to good OS in patients with stage I LUAD. *MUC3A/3B* and *MUC19* were linked to unfavourable OS in patients with stage I and II LUSC, respectively. These findings indicated that *MUC3A/3B* and *MUC19* performed a prognostic function in early-stage NSCLC (Supplementary Tables 2A–2C). High *MUC1* and *MUC3A/3B* mRNA levels correlated with favourable OS, and increased levels of *MUC16* mRNA were considerably linked to unsatisfactory OS in NSCLC patients without chemotherapeutic treatment. Prognosis of *MUC* levels in LUSC and LUAD subsets of patients with or without chemotherapy was not available because the total sample number was low (Supplementary Table 3). Interestingly, increased levels of *MUC1* and *MUC15* mRNA were remarkably related to favourable OS in male patients with LUAD. However, in female patients, *MUC3A/3B*, *MUC8*, and *MUC12* correlated with unfavourable OS. Elevated *MUC16* mRNA levels in male LUAD patients were substantially linked to poor OS (Supplementary Tables 4A–4C).

### 3.4. MUC Gene Alterations in NSCLC

**Figure 6:**
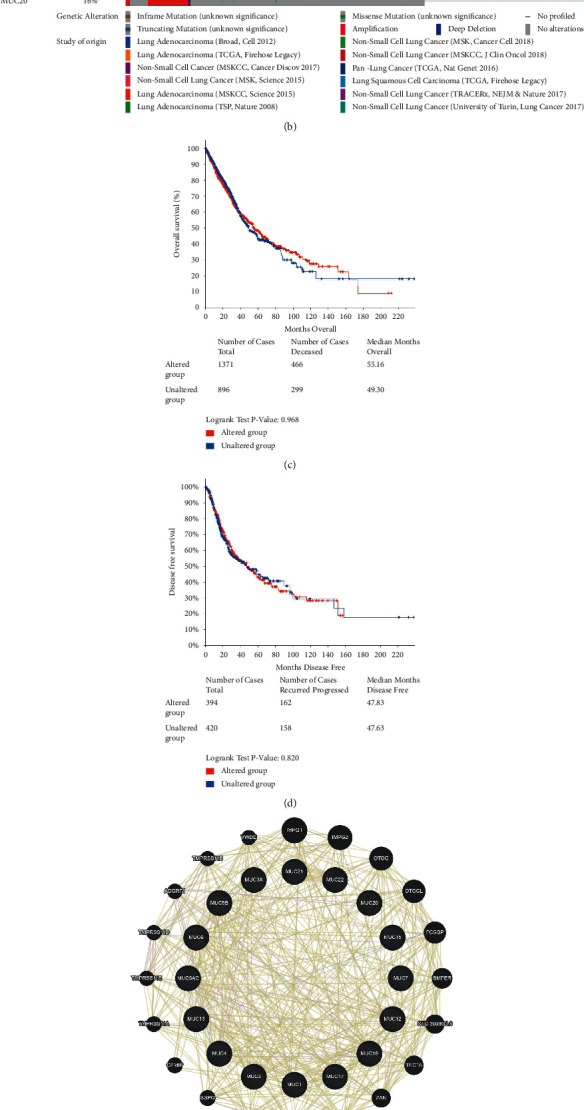
MUC genes alterations and prognosis in NSCLC analysed by cBioPortal for cancer genomics, and the MUC functional network in GeneMANIA. (a) Summary of MUC alterations in NSCLC. (b) Summary of MUC alteration frequencies. (c) OS in NSCLC with and without MUC alterations. (d) DFS in NSCLC with and without MUC alterations. (e) MUC gene-gene interactions. (f) GO and KEGG enrichment analysis result showing the significantly enriched terms. MUC: mucins; NSCLC: non-small-cell lung cancer; OS: overall survival; DFS: disease-free survival; BP: biological process; CC: cellular component; MF: molecular function.

*MUC* genetic alterations that are regularly present in NSCLC patients were studied in the cBioPortal. Thirteen NSCLC datasets were included (Supplementary Table 5). The findings of the investigation indicated that the frequency of *MUC* gene changes, including mutation, fusion, amplification, deep deletion, and multiple changes, range from 0.52% (6/1144) to 81.25% (13/16), among which mutation, amplification, and multiple changes are the most common changes (Figure 6(a)). The percentage of NSCLC-specific MUC genetic changes from 0–28% (*MUC1*, 7%; *MUC2*, 7%; *MUC3A*, 2.5%; *MUC3B*, 0%; *MUC4*, 17%; *MUC5AC*, 0.5%; *MUC5B*, 11%; *MUC6*, 6%; *MUC7*, 3%; *MUC8*, 0.3%; *MUC12*, 4%; *MUC13*, 4%; *MUC15*, 1.2%; *MUC16*, 28%; *MUC17*, 14%; *MUC19*, 2.1%; *MUC20*, 14%; *MUC21*, 2.1%; *MUC2*2, 1.4%; Figure 6(b)), and were mainly mutation, amplification, and deep deletion. These are consistent with the results in Figure 6(a). The samples with at least one gene alteration were included in the altered group, whereas samples without any alterations were included in the unaltered group. The prognostic value of MUC in NSCLC patients with or without changes was investigated, and it was discovered that there was no remarkable relationship between the existence of change, OS and DFS (*P*=0.700 and *P*=0.487, correspondingly; Figures 6(c) and 6(d)). Subsequently, GeneMANIA (an online tool of Cytoscape) was utilized to establish a network of MUCs and relevant functional genes. *MUC3B*, *MUC8*, and *MUC19* were unrecognized in GeneMANIA. The database identified the first 20 genes that were strongly related to MUCs with the default thresholds. In addition, all MUCs had a domain of protein-binding, with MUC4 and MUC16 being colocalized and coexpressed within the cell (Figure 6(e)).

### 3.5. MUC Gene Enrichment Analysis in NSCLC

The functions of *MUC* genes were analysed with the DAVID. Twelve GO terms were found to be enriched (Figure 6(f)). An enrichment in MUC proteins was found in the biological processes (BP) involving O-glycan processing and maintenance of the gastrointestinal epithelium. MUC acts as an extracellular matrix structural constituent, and its lubricant activity is the molecular function (MF) associated with it. The Golgi lumen, extracellular exosome, extracellular space, extracellular region, apical plasma membrane, integral component of membrane, vesicle, and mucus layer were the cellular components (CC) associated with MUC. The salivary secretion pathway was enriched for MUCs in KEGG.

## 4. Discussion

### 4.1. MUCs in the Human Airway

The susceptibility of inherited genes involved in lung cancer and environmental carcinogens are important factors in lung cancer aetiology. Differential expression of all the factors demonstrates population heterogeneity. MUCs are glycoproteins synthesized by mucosal epithelial cells. The expression of MUCs promotes cell invasion and metastasis and is regarded as a risk factor, demonstrating a poor prognosis. Lung cancer is among the most fatal tumours globally, and LUAD is the most prevalent subtype. Histological classification and early diagnosis are required for individualised treatments [24]. Various cancer treatment strategies, including molecular targeted therapy, stem cells, vaccines, oncolytic immunotherapy, and genetic therapy, are regarded as promising modalities, especially for patients whose lung cancer is at an advanced stage. Specific biomarkers and accurate diagnosis act as decisive factors for the appropriate treatment. However, the mechanism of MUC expression in lung cancer is yet to be systematically elucidated. Here, we studied the expression of 19 MUC proteins (MUC1, MUC2, MUC3A, MUC3B, MUC4, MUC5AC, MUC5B, MUC6, MUC7, MUC8, MUC12, MUC13, MUC15, MUC16, MUC17, MUC19, MUC20, MUC21, and MUC22) that are observed in the human airway, along with the MUC regulatory network in patients with NSCLC using bioinformatics analysis.

### 4.2. MUCs in NSCLC

It was shown that MUC1, the most highly expressed MUC in lung cancer, was expressed specifically in invasive lepidic predominant adenocarcinoma (LPA) [25]. Moreover, the depolarization of cells impacted MUC1 expression in lung cancer progression [11]. Besides, the specificity and efficacy of the prostate stem cell antigen (PSCA)- and MUC1-targeting chimeric antigen receptor (CAR) T cells against NSCLC cell lines in vitro were confirmed [26]. It is also known that MUC1-C ⟶ PD-L1 signaling promotes the inhibition of CD8 T cell activation [27]. Therefore, MUC1 would be a highly attractive antigen for the development of effective anticancer vaccines and a potential molecular target for reprogramming the tumour microenvironment. Our study demonstrated that the *MUC1* mRNA expression was remarkably lower in LUSC as opposed to that in normal lung specimens, and differential *MUC1* expression was observed during the tumour stage progressing from I to IV. Increased *MUC1* and *MUC15* mRNA levels were linked to favourable OS in LUAD patients.

MUC2 and MUC6 have been related to lymph node metastasis in LUAD patients [28]. Additionally, DNA hypomethylation was illustrated to perform an instrumental function in MUC3A expression in carcinomas [29]. Our study found increased *MUC2* and *MUC3A* mRNA levels linked to unfavourable OS in LUAD patients.

MUC4 expression is independent of mucus secretion in both normal human airways and carcinomas before epithelial differentiation [30]. MUC4 correlated with a better OS; MUC4 seemed to play a potential protective role in early-stage LUAD [31, 32]. MUC4-positive LUAD mediated by the human epidermal growth factor receptor (HER)2 signaling pathway might be a distinct LUAD subtype in patients with poor outcomes associated with smoking [33]. Our results showed that the *MUC4* mRNA expression level in LUAD was considerably elevated as opposed to normal lung specimens. However, mRNA levels of *MUC4* were not substantially linked to OS and DFS in patients with NSCLC. Owing to the conflicting evidence, further experiments are required to examine the molecular mechanism of whether MUC4 is oncogenic or tumour suppressive.

MUC5AC and MUC5B have been used as specific markers to detect central type LUAD and mucinous LUAD [34]. MUC5AC was found to be a significant determinant of a poor prognosis, especially in KRAS-mutant tumours [35]. In ALK + lung cancer, there is a higher incidence of MUC1 and MUC5AC cytoplasmic expression, which, combined with a paucity of MUC2 and MUC6 expression, could lead to the biological aggressiveness of ALK + cancer [36]. In a recent study, it was observed that histological subgroups were associated with ALK, KRAS, and MET mutations, and with immunohistochemical reactivity of MUC1, MUC5AC, and MUC6 among the Chinese population [36]. MUC production independently served as a prognostic indicator for the epidermal growth factor receptor (EGFR)-mutant LUAD that was characterised by negative MUC5AC-staining and positive MUC5B-staining [37]. Overexpressed MUC5AC in genetically engineered mouse LUAD tissues was associated with poor survival in comparison with normal lung tissues [38]. However, in our study, the different MUC5AC expression has not been observed between the tumour and normal tissues as well as between diverse tumour stages. Nonetheless, the elevated *MUC5AC* mRNA expression level was substantially linked to unfavourable DFS in patients with LUAD. Further research will help to clarify the exact role of the MUC5AC gene subtype.

A combination of high expression of MUC5B with thyroid transcription factor (TTF)-1 negative cells was a valuable marker to prophesize a poor OS of patients with LUAD compared with that of patients with LUSC [39]. Besides, it has been shown that the polymorphism in the MUC5B promoter can act as a predictive marker of OS in NSCLC patients receiving radiotherapy [40]. In this investigation, the *MUC5B* mRNA expression level was almost twice as higher in LUAD compared to that in normal lungs and changed significantly across various tumour stages. Especially, the expression level of MUC5B in stage IV was almost as twice in stage II.

MUC6 was shown to be upmodulated in the peritumoral epithelial tissues. Besides, the expressions of MUC8, MUC5AC, and MUC4 were reduced in NSCLC [41]. MUC7 was related to cell differentiation in smoke-induced lung cancer [42]. In this study, the elevated *MUC8* mRNA expression level in LUAD patients was notably linked to OS.

Mutations in MUC12 have been observed at higher frequencies in the samples of familial lung cancer samples and lung cancer tissue, compared with those in the healthy population [43]. In this study, an increased expression of *MUC12*, *MUC13*, *MUC16*, and *MUC17* mRNAs was substantially linked to unfavourable OS in patients with LUAD in the KM plotter. However, the result cannot be wholly proved in GEPIA; only *MUC2*, *MUC12*, and *MUC16* could be associated with alteration in OS using this server. This might be attributed to the fact that the GEPIA database has a lower sample size.

MUC16, which is primarily expressed on the human goblet cell surface, demonstrates overexpression in patients with NSCLC and is often correlated with an unfavourable prognosis. MUC16 performs a meaningful function in metastasis and tumourigenesis in lung cancer by regulating TSPYL5 through JAK2/STAT3/GR [44]. A MUC16-mutant was resistant to matrix-metalloproteases (MMPs) that were released by LUAD cells. Furthermore, LUAD with both MMP- and MUC16-resistant mutant expression had an unfavourable prognosis [45]. The overexpression of MUC16 was correlated with familial lung cancer, air pollution produced by coal indoor, higher metastasis, and an advanced stage. High MUC16 expression contributed to the capacity of lung cancer cells to proliferate, invade, resist chemotherapy, and migrate in experiments analyzing cell behaviour. However, the results demonstrate variations among cell lines [46]. This research illustrated that the expression of *MUC16* mRNA was higher in LUAD in contrast to that in normal tissues. *MUC16* mRNA was remarkably linked to poor OS in patients with NSCLC and LUAD. Enhanced *MUC16* mRNA expression was strongly linked to adverse OS in patients with smoking history, in those without chemotherapeutic treatment, and in males.

The examination of the role of MUC20 in NSCLC is still incomplete. However, in endometrial cancer, MUC20 overexpression drives tumourigenesis, predicts poor survival [47], and EGF-induced malignant phenotypes were enhanced by activating the EGFR/STAT3 pathway [48]. Large-scale genomic dataset analyses demonstrated that the synergistic effect of MUC4, MUC16, and MUC20 was linked to a statistically significant reduction in OS and elevated HR in colon, stomach, and pancreatic cancers [9]. This research illustrated that the expression level of *MUC20* mRNA was substantially elevated in NSCLC (both LUSC and LUAD) in contrast to that in normal lung samples, but exhibited no link to DFS or OS in NSCLC.

MUC21 is a novel transmembrane MUC that could be used as a negative immunohistochemical marker to differentiate mesothelioma from LUAD [49, 50]. MUC21 could be a promising biomarker with potential diagnostic and therapeutic applications for LUAD showing cell incohesiveness [10]. MUC22 was shown to independently function as a specific prognostic indicator of OS in patients with LUSC [51].

Analyses of the link between *MUC* mRNA expression and DFS/OS in NSCLC patients were performed by using two public datasets exhibited similar results. However, the observations on MUC expression were not completely consistent among the different datasets. This could be attributed to the fact that GEPIA has a smaller sample size compared to that in the KM Plotter. It suggests that larger sample sizes and more detailed oncogenic driver-based subgroups should be considered in the future to improve the quality of the analysis.

The link between MUC mRNA and a variety of clinical-pathological features was investigated. We found that *MUC15* was linked to favourable OS in LUAD male patients and with smoking activity. In contrast, *MUC12* was considerably related to unsatisfactory OS in LUAD patients with a smoking history, stage I or II tumours, and being female.

Mutations in MUCs could be linked to tumourigenesis and cancer development, and may act as potential tumour suppressors and genic biomarkers. Different types of alterations that are commonly observed were analysed in MUCs with NSCLC. High enrichment of amplification events in TCGA LUSC compared to other datasets suggests a role of MUC mutations on LUSC, especially those in MUC1, MUC4, and MUC20. However, the observed alterations did not have any correlation with OS or DFS. The results suggest that these gene mutations may not directly affect NSCLC prognosis. Additionally, MUC proteins were evaluated with network analysis to examine the potential molecular mechanisms of MUC in NSCLC. *MUC* genes were primarily enriched in the O-glycan processing and maintenance of gastrointestinal epithelium pathways, highlighting its role as a potential target for anti-NSCLC therapeutics, especially for MUC-producing LUAD.

### 4.3. Prognostic Value of MUCs in NSCLC

The study showed that the elevated expression of MUC1 and MUC15 was considerably linked to favourable OS in patients with NSCLC, particularly in patients with LUAD. The elevated MUC8, MUC12, and MUC16 expression levels were substantially related to poor OS in patients with LUAD (Supplementary Table 6). MUC4 and MUC16 were colocalized and coexpressed within the cell. The study suggested the clinical personal heterogeneity and NSCLC signaling complexity, and highlight the combination of associated MUCs as a potential tool for the determination of prognosis and use in molecular targeted therapy for patients with lung cancer. More research will be required to investigate MUC protein expression in various oncogenic driver subtypes. This study will aid in further evaluation of the molecular mechanisms of MUCs in NSCLC as well as in the exploration of the potential of MUC-based therapeutic targets for NSCLC treatment.

## Data Availability

Publicly available datasets were used for this study. The analysis of gene expression profiling and prognosis was performed with the GEPIA database (https://gepia.cancer-pku.cn). Protein expression levels were obtained from HPA (https://www.proteinatlas.org/). The prognostic analysis was performed with the KM plotter tool (https://www.kmplot.com). The analysis of gene alteration frequency was performed using the cBioPortal for cancer genomics (https://www.cbioportal.org). Gene correlation analysis was conducted with GeneMANIA (https://www.genemania.org). The functional annotation and pathway enrichment analysis were performed with the DAVID (https://david.ncifcrf.gov/).

## Supplementary Materials

Supplementary Materials Supplementary Figure 1: representive IHC images of MUC protein expression in normal, LUAD, and LUSC tissue in the HPA (MUC20 and MUC22 were not available). IHC: immunohistochemistry; Ab: antibody; MUC: mucins; LUAD: lung adenocarcinoma; LUSC: lung squamous cell carcinoma; HPA: Human Protein Atlas. Supplementary Table 1A: correlation between *MUC* mRNA levels and OS in patients with NSCLC with smoking statuses. Significant results are marked in bold. MUCs: mucins; OS: overall survival; NSCLC: non-small-cell lung cancer; HR: hazard ratio; CI: confidence intervals. Supplementary Table 1B: correlation between *MUC* mRNA levels and OS in patients with LUAD with smoking statuses. Significant results are marked in bold. MUCs: mucins; OS: overall survival; LUAD: lung adenocarcinoma; HR: hazard ratio; CI: confidence intervals. Supplementary Table 1C: correlation between *MUC* mRNA levels and OS in patients with LUSC with smoking statuses. Significant results are marked in bold. MUCs: mucins; OS: overall survival; LUSC: lung squamous cell carcinoma; HR: hazard ratio; CI: confidence intervals. Supplementary Table 2A: correlation between *MUC* mRNA levels and OS in patients with NSCLC at different clinical stages. Significant results are marked in bold. MUC: mucins; OS: overall survival; NSCLC: non-small-cell lung cancer; HR: hazard ratio; CI: confidence intervals. Supplementary Table 2B: correlation between *MUC* mRNA levels and OS in patients with LUAD at different clinical stages. Significant results are marked in bold. MUC: mucins; OS: overall survival; LUAD: lung adenocarcinoma; HR: hazard ratio; CI: confidence intervals. Supplementary Table 2C: correlation between *MUC* mRNA levels and OS in patients with LUSC at different clinical stages. Significant results are marked in bold. MUC: mucins; OS: overall survival; LUSC: lung squamous cell carcinoma; HR: hazard ratio; CI: confidence intervals. Supplementary Table 3: correlation between *MUC* mRNA levels and OS in patients with NSCLC according to their chemotherapy status. Significant results are marked in bold. MUC, mucins; OS, overall survival; NSCLC, non-small cell lung cancer; HR, hazard ratio; CI, confidence intervals. Supplementary Table 4A: correlation between *MUC* mRNA level and OS in patients of different sex with NSCLC. Significant results are marked in bold. MUCs, mucins; OS, overall survival; NSCLC, non-small-cell lung cancer; HR, hazard ratio; CI: confidence intervals. Supplementary Table 4B: correlation between *MUC* mRNA levels and OS in patients of different sex with LUAD. Significant results are marked in bold. MUCs: mucins; OS: overall survival; LUAD: lung adenocarcinoma; HR: hazard ratio; CI: confidence intervals. Supplementary Table 4C: correlation between *MUC* mRNA levels and OS in patients of different sex with LUSC. Significant results are marked in bold. MUCs: mucins; OS: overall survival; LUSC: lung squamous cell carcinoma; HR: hazard ratio; CI: confidence intervals. Supplementary Table 5: the information of the 13 NSCLC datasets. Supplementary Table 6: the expression and prognostic value of MUCs in NSCLC. MUCs: mucins; NSCLC: non-small-cell lung cancer.

## References

[1] Zeng Z., Yang F., Wang Y. (2020). Significantly different immunoscores in lung adenocarcinoma and squamous cell carcinoma and a proposal for a new immune staging system. *OncoImmunology*.

[2] Arbour K. C., Riely G. J. (2019). Systemic therapy for locally advanced and metastatic non-small cell lung cancer: a review. *JAMA*.

[3] Moniaux N., Andrianifahanana M., Brand R. E., Batra S. K. (2004). Multiple roles of mucins in pancreatic cancer, a lethal and challenging malignancy. *British Journal of Cancer*.

[4] Felder M., Kapur A., Gonzalez-Bosquet J. (2014). MUC16 (CA125): tumor biomarker to cancer therapy, a work in progress. *Molecular Cancer*.

[5] Aithal A., Rauth S., Kshirsagar P. (2018). MUC16 as a novel target for cancer therapy. *Expert Opinion on Therapeutic Targets*.

[6] Matsuyama T., Ishikawa T., Mogushi K. (2010). MUC12 mRNA expression is an independent marker of prognosis in stage II and stage III colorectal cancer. *International Journal of Cancer*.

[7] Dai W., Liu J., Liu B., Li Q., Sang Q., Li Y. Y. (2020). Systematical analysis of the cancer genome atlas database reveals EMCN/MUC15 combination as a prognostic signature for gastric cancer. *Frontiers in Molecular Biosciences*.

[8] Maher D. M., Gupta B. K., Nagata S., Jaggi M., Chauhan S. C. (2011). Mucin 13: structure, function, and potential roles in cancer pathogenesis. *Molecular Cancer Research*.

[9] Jonckheere N., Van Seuningen I. (2018). Integrative analysis of the cancer genome atlas and cancer cell lines encyclopedia large-scale genomic databases: MUC4/MUC16/MUC20 signature is associated with poor survival in human carcinomas. *Journal of Translational Medicine*.

[10] Yoshimoto T., Matsubara D., Soda M. (2019). Mucin 21 is a key molecule involved in the incohesive growth pattern in lung adenocarcinoma. *Cancer Science*.

[11] Lakshmanan I., Ponnusamy M. P., Macha M. A. (2015). Mucins in lung cancer: diagnostic, prognostic, and therapeutic implications. *Journal of Thoracic Oncology*.

[12] Tang Z., Li C., Kang B., Gao G., Li C., Zhang Z. (2017). GEPIA: a web server for cancer and normal gene expression profiling and interactive analyses. *Nucleic Acids Research*.

[13] Thul P. J., Åkesson L., Wiking M. (2017). A subcellular map of the human proteome. *Science (New York, N.Y)*.

[14] Uhlén M., Fagerberg L., Hallström B. M. (2015). Proteomics. Tissue-based map of the human proteome. *Science (New York, N.Y)*.

[15] Uhlen M., Zhang C., Lee S. (2017). A pathology atlas of the human cancer transcriptome. *Science (New York, N.Y)*.

[16] Nagy Á., Lánczky A., Menyhárt O., Győrffy B. (2018). Validation of miRNA prognostic power in hepatocellular carcinoma using expression data of independent datasets. *Scientific Reports*.

[17] Gyorffy B., Lánczky A., Szállási Z. (2012). Implementing an online tool for genome-wide validation of survival-associated biomarkers in ovarian-cancer using microarray data from 1287 patients. *Endocrine-Related Cancer*.

[18] Győrffy B., Surowiak P., Budczies J., Lánczky A. (2013). Online survival analysis software to assess the prognostic value of biomarkers using transcriptomic data in non-small-cell lung cancer. *PLoS One*.

[19] Cerami E., Gao J., Dogrusoz U. (2012). The cBio cancer genomics portal: an open platform for exploring multidimensional cancer genomics data. *Cancer Discovery*.

[20] Gao J., Aksoy B. A., Dogrusoz U. (2013). Integrative analysis of complex cancer genomics and clinical profiles using the cBioPortal. *Science Signaling*.

[21] Warde-Farley D., Donaldson S. L., Comes O. (2010). The GeneMANIA prediction server: biological network integration for gene prioritization and predicting gene function. *Nucleic Acids Research*.

[22] Huang D. W., Sherman B. T., Lempicki R. A. (2009). Systematic and integrative analysis of large gene lists using DAVID bioinformatics resources. *Nature Protocols*.

[23] Huang D. W., Sherman B. T., Lempicki R. A. (2009). Bioinformatics enrichment tools: paths toward the comprehensive functional analysis of large gene lists. *Nucleic Acids Research*.

[24] Arroyo M., Larrosa R., Gómez-Maldonado J., Cobo M., Claros M. G., Bautista R. (2020). Expression-based, consistent biomarkers for prognosis and diagnosis in lung cancer. *Clinical and Translational Oncology*.

[25] Duruisseaux M., Antoine M., Rabbe N. (2017). Lepidic predominant adenocarcinoma and invasive mucinous adenocarcinoma of the lung exhibit specific mucin expression in relation with oncogenic drivers. *Lung Cancer*.

[26] Wei X., Lai Y., Li J. (2017). PSCA and MUC1 in non-small-cell lung cancer as targets of chimeric antigen receptor T cells. *OncoImmunology*.

[27] Bouillez A., Adeegbe D., Jin C. (2017). MUC1-C promotes the suppressive immune microenvironment in non-small cell lung cancer. *OncoImmunology*.

[28] Nishiumi N., Abe Y., Inoue Y. (2003). Use of 11p15 mucins as prognostic factors in small adenocarcinoma of the lung. *Clinical Cancer Research: An Official Journal of the American Association for Cancer Research*.

[29] Kitamoto S., Yamada N., Yokoyama S., Houjou I., Higashi M., Yonezawa S. (2010). Promoter hypomethylation contributes to the expression of MUC3A in cancer cells. *Biochemical and Biophysical Research Communications*.

[30] Gosselin B., Buisine M. P., Devisme L. (2001). Normal respiratory mucosa, precursor lesions and lung carcinomas: differential expression of human mucin genes. *Frontiers in Bioscience*.

[31] Kwon K. Y., Ro J. Y., Singhal N. (2007). MUC4 expression in non-small cell lung carcinomas: relationship to tumor histology and patient survival. *Archives of Pathology & Laboratory Medicine*.

[32] Majhi P. D., Lakshmanan I., Ponnusamy M. P. (2013). Pathobiological implications of MUC4 in non-small-cell lung cancer. *Journal of Thoracic Oncology*.

[33] Rokutan-Kurata M., Yoshizawa A., Sumiyoshi S. (2017). Lung adenocarcinoma with MUC4 expression is associated with smoking status, HER2 protein expression, and poor prognosis: clinicopathologic analysis of 338 cases. *Clinical Lung Cancer*.

[34] Kim Y. K., Shin D. H., Kim K. B. (2015). MUC5AC and MUC5B enhance the characterization of mucinous adenocarcinomas of the lung and predict poor prognosis. *Histopathology*.

[35] Bauer A. K., Umer M., Richardson V. L. (2018). Requirement for MUC5AC in KRAS-dependent lung carcinogenesis. *JCI insight*.

[36] Shang G., Jin Y., Zheng Q. (2019). Histology and oncogenic driver alterations of lung adenocarcinoma in Chinese. *American journal of cancer research*.

[37] Wakejima R., Inamura K., Ninomiya H. (2020). Mucinous lung adenocarcinoma, particularly referring to EGFR-mutated mucinous adenocarcinoma. *Pathology International*.

[38] Lakshmanan I., Rachagani S., Hauke R. (2016). MUC5AC interactions with integrin *β*4 enhances the migration of lung cancer cells through FAK signaling. *Oncogene*.

[39] Nagashio R., Ueda J., Ryuge S. (2015). Diagnostic and prognostic significances of MUC5B and TTF-1 expressions in resected non-small cell lung cancer. *Scientific Reports*.

[40] Yang J., Xu T., Gomez D. R. (2017). The pulmonary fibrosis associated MUC5B promoter polymorphism is prognostic of the overall survival in patients with non-small cell lung cancer (NSCLC) receiving definitive radiotherapy. *Translational oncology*.

[41] López-Ferrer A., Curull V., Barranco C. (2001). Mucins as differentiation markers in bronchial epithelium. Squamous cell carcinoma and adenocarcinoma display similar expression patterns. *American Journal of Respiratory Cell and Molecular Biology*.

[42] Woenckhaus M., Klein-Hitpass L., Grepmeier U. (2006). Smoking and cancer-related gene expression in bronchial epithelium and non-small-cell lung cancers. *The Journal of Pathology*.

[43] Kanwal M., Ding X. J., Ma Z. H. (2018). Characterization of germline mutations in familial lung cancer from the Chinese population. *Gene*.

[44] Lakshmanan I., Salfity S., Seshacharyulu P. (2017). MUC16 regulates TSPYL5 for lung cancer cell growth and chemoresistance by suppressing p53. *Clinical Cancer Research*.

[45] Patel J. S., Callahan B. M., Chobrutskiy B. I., Blanck G. (2019). Matrix-metalloprotease resistant mucin-16 (MUC16) peptide mutants represent a worse lung adenocarcinoma outcome. *Proteomics-Clinical Applications*.

[46] Chen Y., Huang Y., Kanwal M. (2019). MUC16 in non-small cell lung cancer patients affected by familial lung cancer and indoor air pollution: clinical characteristics and cell behaviors. *Translational Lung Cancer Research*.

[47] Zheng F., Yu H., Lu J. (2019). High expression of MUC20 drives tumorigenesis and predicts poor survival in endometrial cancer. *Journal of Cellular Biochemistry*.

[48] Chen C. H., Wang S. W., Chen C. W. (2013). MUC20 overexpression predicts poor prognosis and enhances EGF-induced malignant phenotypes via activation of the EGFR-STAT3 pathway in endometrial cancer. *Gynecologic Oncology*.

[49] Itoh Y., Kamata-Sakurai M., Denda-Nagai K. (2007). Identification and expression of human epiglycanin/MUC21: a novel transmembrane mucin. *Glycobiology*.

[50] Kai Y., Amatya V. J., Kushitani K. (2019). Mucin 21 is a novel, negative immunohistochemical marker for epithelioid mesothelioma for its differentiation from lung adenocarcinoma. *Histopathology*.

[51] Xu F., Lin H., He P. (2020). A TP53-associated gene signature for prediction of prognosis and therapeutic responses in lung squamous cell carcinoma. *OncoImmunology*.

