# Supplementary figures and images for: Expression of Mucin Family Proteins in Non-Small-Cell Lung Cancer and its Role in Evaluation of Prognosis
Source: J Oncol. 2022 Aug 26;2022:4181658. doi: 10.1155/2022/4181658 (PMC9439898; doi:10.1155/2022/4181658)

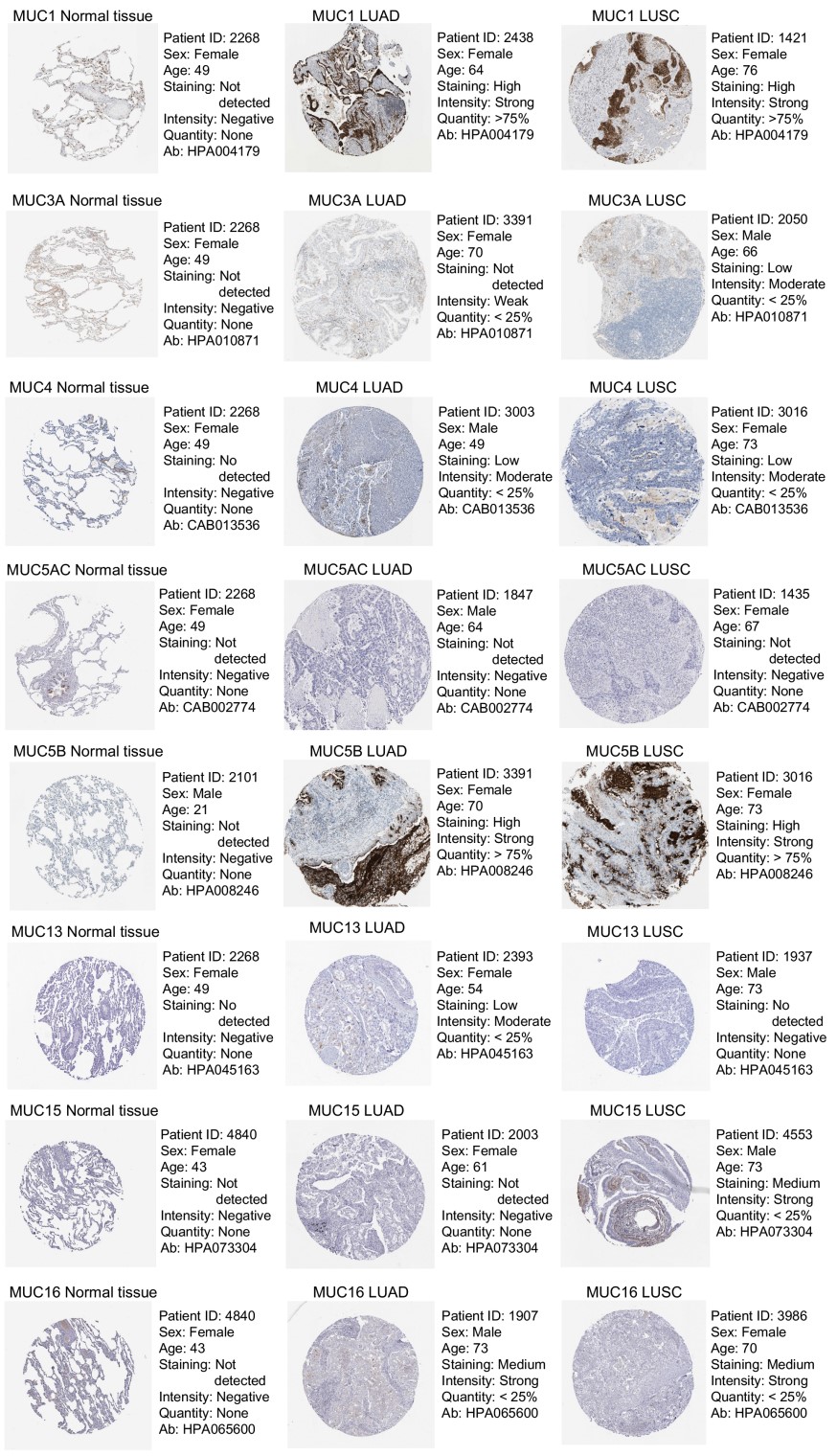

Supplement: Supplementary Materials — Supplementary Figure 1: representive IHC images of MUC protein expression in normal, LUAD, and LUSC tissue in the HPA (MUC20 and MUC22 were not available). IHC: immunohistochemistry; Ab: antibody; MUC: mucins; LUAD: lung adenocarcinoma; LUSC: lung squamous cell carcinoma; HPA: Human Protein Atlas. Supplementary Table 1A: correlation between MUC mRNA levels and OS in patients with NSCLC with smoking statuses. Significant results are marked in bold. MUCs: mucins; OS: overall survival; NSCLC: non-small-cell lung cancer; HR: hazard ratio; CI: confidence intervals. Supplementary Table 1B: correlation between MUC mRNA levels and OS in patients with LUAD with smoking statuses. Significant results are marked in bold. MUCs: mucins; OS: overall survival; LUAD: lung adenocarcinoma; HR: hazard ratio; CI: confidence intervals. Supplementary Table 1C: correlation between MUC mRNA levels and OS in patients with LUSC with smoking statuses. Significant results are marked in bold. MUCs: mucins; OS: overall survival; LUSC: lung squamous cell carcinoma; HR: hazard ratio; CI: confidence intervals. Supplementary Table 2A: correlation between MUC mRNA levels and OS in patients with NSCLC at different clinical stages. Significant results are marked in bold. MUC: mucins; OS: overall survival; NSCLC: non-small-cell lung cancer; HR: hazard ratio; CI: confidence intervals. Supplementary Table 2B: correlation between MUC mRNA levels and OS in patients with LUAD at different clinical stages. Significant results are marked in bold. MUC: mucins; OS: overall survival; LUAD: lung adenocarcinoma; HR: hazard ratio; CI: confidence intervals. Supplementary Table 2C: correlation between MUC mRNA levels and OS in patients with LUSC at different clinical stages. Significant results are marked in bold. MUC: mucins; OS: overall survival; LUSC: lung squamous cell carcinoma; HR: hazard ratio; CI: confidence intervals. Supplementary Table 3: correlation between MUC mRNA levels and OS in patients with NSCLC acco [file 4181658.f1.zip › Supplementary Figure 1.jpg]
