# Supplementary material for: Expression of Mucin Family Proteins in Non-Small-Cell Lung Cancer and its Role in Evaluation of Prognosis
Source: J Oncol. 2022 Aug 26;2022:4181658. doi: 10.1155/2022/4181658 (PMC9439898; doi:10.1155/2022/4181658)
Supplement: Supplementary Materials — Supplementary Figure 1: representive IHC images of MUC protein expression in normal, LUAD, and LUSC tissue in the HPA (MUC20 and MUC22 were not available). IHC: immunohistochemistry; Ab: antibody; MUC: mucins; LUAD: lung adenocarcinoma; LUSC: lung squamous cell carcinoma; HPA: Human Protein Atlas. Supplementary Table 1A: correlation between MUC mRNA levels and OS in patients with NSCLC with smoking statuses. Significant results are marked in bold. MUCs: mucins; OS: overall survival; NSCLC: non-small-cell lung cancer; HR: hazard ratio; CI: confidence intervals. Supplementary Table 1B: correlation between MUC mRNA levels and OS in patients with LUAD with smoking statuses. Significant results are marked in bold. MUCs: mucins; OS: overall survival; LUAD: lung adenocarcinoma; HR: hazard ratio; CI: confidence intervals. Supplementary Table 1C: correlation between MUC mRNA levels and OS in patients with LUSC with smoking statuses. Significant results are marked in bold. MUCs: mucins; OS: overall survival; LUSC: lung squamous cell carcinoma; HR: hazard ratio; CI: confidence intervals. Supplementary Table 2A: correlation between MUC mRNA levels and OS in patients with NSCLC at different clinical stages. Significant results are marked in bold. MUC: mucins; OS: overall survival; NSCLC: non-small-cell lung cancer; HR: hazard ratio; CI: confidence intervals. Supplementary Table 2B: correlation between MUC mRNA levels and OS in patients with LUAD at different clinical stages. Significant results are marked in bold. MUC: mucins; OS: overall survival; LUAD: lung adenocarcinoma; HR: hazard ratio; CI: confidence intervals. Supplementary Table 2C: correlation between MUC mRNA levels and OS in patients with LUSC at different clinical stages. Significant results are marked in bold. MUC: mucins; OS: overall survival; LUSC: lung squamous cell carcinoma; HR: hazard ratio; CI: confidence intervals. Supplementary Table 3: correlation between MUC mRNA levels and OS in patients with NSCLC acco [file 4181658.f1.zip › Supplementary_Table_1.DOC]

**Supplementary Table 1A**. Correlation between MUC mRNA level and OS in NSCLC patients with smoking status.

| MUC family | Smoking status | Cases | HR | 95% CI | P-value |
| --- | --- | --- | --- | --- | --- |
| MUC1 | Never smoked | 205 | 0.72 | 0.41 - 1.26 | 0.24 |
|  | Smoked | 820 | 0.76 | 0.62 - 0.94 | **0.01** |
| MUC2 | Never smoked | 205 | 0.82 | 0.47 - 1.43 | 0.48 |
|  | Smoked | 820 | 1.31 | 1.07 - 1.62 | **0.0099** |
| MUC3A | Never smoked | 205 | 1.9 | 1.07 - 3.36 | **0.026** |
|  | Smoked | 820 | 1.12 | 0.91 - 1.38 | 0.27 |
| MUC3B | Never smoked | 205 | 1.9 | 1.07 - 3.36 | **0.026** |
|  | Smoked | 820 | 1.12 | 0.91 - 1.38 | 0.27 |
| MUC4 | Never smoked | 205 | 0.86 | 0.49 - 1.5 | 0.6 |
|  | Smoked | 820 | 1.09 | 0.89 - 1.34 | 0.4 |
| MUC5AC | Never smoked | 205 | 1.02 | 0.58 - 1.77 | 0.95 |
|  | Smoked | 820 | 1.22 | 0.99 - 1.5 | 0.063 |
| MUC5B | Never smoked | 205 | 1.26 | 0.72 - 2.2 | 0.42 |
|  | Smoked | 820 | 1.09 | 0.88 - 1.34 | 0.43 |
| MUC6 | Never smoked | 205 | 1.35 | 0.77 - 2.37 | 0.29 |
|  | Smoked | 820 | 1.29 | 1.05 - 1.58 | **0.017** |
| MUC7 | Never smoked | 205 | 0.72 | 0.41 - 1.27 | 0.25 |
|  | Smoked | 820 | 1.06 | 0.86 - 1.3 | 0.58 |
| MUC8 | Never smoked | 205 | 1.42 | 0.82 - 2.49 | 0.21 |
|  | Smoked | 820 | 1.52 | 1.23 - 1.87 | **7.3e - 05** |
| MUC12 | Never smoked | 205 | 2.09 | 0.89 - 4.88 | 0.082 |
|  | Smoked | 820 | 1.78 | 1.18 - 2.68 | **0.0056** |
| MUC13 | Never smoked | 205 | 1.01 | 0.58 - 1.75 | 0.99 |
|  | Smoked | 820 | 1.15 | 0.94 - 1.41 | 0.18 |
| MUC15 | Never smoked | 205 | 0.4 | 0.17 - 0.94 | **0.029** |
|  | Smoked | 820 | 0.44 | 0.28 - 0.68 | **0.00015** |
| MUC16 | Never smoked | 205 | 1.33 | 0.76 - 2.34 | 0.32 |
|  | Smoked | 820 | 1.47 | 1.19 - 1.81 | **0.00027** |
| MUC17 | Never smoked | 205 | 0.7 | 0.31 - 1.58 | 0.39 |
|  | Smoked | 820 | 1.3 | 0.87 - 1.95 | 0.2 |
| MUC19 | Never smoked | 141 | 0.54 | 0.23 - 1.24 | 0.14 |
|  | Smoked | 300 | 1.13 | 0.75 - 1.69 | 0.57 |
| MUC20 | Never smoked | 205 | 1.13 | 0.51 - 2.53 | 0.76 |
|  | Smoked | 820 | 1.13 | 0.75 - 1.7 | 0.55 |

Significant results are marked in bold. MUC, mucins; OS, overall survival; NSCLC, non-small cell lung cancer; HR, hazard ratio; CI, confidence intervals.

**Supplementary Table 1B**. Correlation between MUC mRNA level and OS in LUAD patients with smoking status.

| MUC family | Smoking status | Cases | HR | 95% CI | P-value |
| --- | --- | --- | --- | --- | --- |
| MUC1 | Never smoked | 143 | 1.09 | 0.49 - 2.42 | 0.84 |
|  | Smoked | 246 | 0.81 | 0.51 - 1.3 | 0.38 |
| MUC2 | Never smoked | 143 | 0.46 | 0.2 - 1.09 | 0.07 |
|  | Smoked | 246 | 1.08 | 0.67 - 1.72 | 0.76 |
| MUC3A | Never smoked | 143 | 1 | 0.45 - 2.25 | 0.99 |
|  | Smoked | 246 | 1.18 | 0.74 - 1.89 | 0.48 |
| MUC3B | Never smoked | 143 | 1 | 0.45 - 2.25 | 0.99 |
|  | Smoked | 246 | 1.18 | 0.74 - 1.89 | 0.48 |
| MUC4 | Never smoked | 143 | 1.32 | 0.58 - 2.97 | 0.51 |
|  | Smoked | 246 | 1.51 | 0.94 - 2.42 | 0.083 |
| MUC5AC | Never smoked | 143 | 0.86 | 0.39 - 1.92 | 0.72 |
|  | Smoked | 246 | 1.02 | 0.64 - 1.62 | 0.94 |
| MUC5B | Never smoked | 143 | 1.29 | 0.58 - 2.89 | 0.53 |
|  | Smoked | 246 | 1.37 | 0.85 - 2.2 | 0.2 |
| MUC6 | Never smoked | 143 | 1.22 | 0.55 - 2.71 | 0.63 |
|  | Smoked | 246 | 1.01 | 0.63 - 1.61 | 0.97 |
| MUC7 | Never smoked | 143 | 0.72 | 0.32 - 1.63 | 0.43 |
|  | Smoked | 246 | 0.85 | 0.53 - 1.36 | 0.5 |
| MUC8 | Never smoked | 143 | 1.88 | 0.83 - 4.24 | 0.12 |
|  | Smoked | 246 | 1.45 | 0.9 - 2.31 | 0.12 |
| MUC12 | Never smoked | 140 | 1.96 | 0.83 - 4.62 | 0.12 |
|  | Smoked | 231 | 2.74 | 1.63 - 4.61 | **7.5e - 05** |
| MUC13 | Never smoked | 143 | 0.64 | 0.28 - 1.47 | 0.29 |
|  | Smoked | 246 | 1.1 | 0.69 - 1.76 | 0.68 |
| MUC15 | Never smoked | 140 | 0.46 | 0.2 - 1.1 | 0.073 |
|  | Smoked | 231 | 0.39 | 0.23 - 0.65 | **0.00023** |
| MUC16 | Never smoked | 143 | 1.23 | 0.55 - 2.74 | 0.62 |
|  | Smoked | 246 | 2.63 | 1.6 - 4.33 | **7.8e - 05** |
| MUC17 | Never smoked | 140 | 0.75 | 0.33 - 1.72 | 0.5 |
|  | Smoked | 231 | 1.43 | 0.88 - 2.32 | 0.15 |
| MUC19 | Never smoked | 140 | 0.48 | 0.2 - 1.13 | 0.086 |
|  | Smoked | 231 | 0.77 | 0.47 - 1.26 | 0.3 |
| MUC20 | Never smoked | 140 | 1.05 | 0.46 - 2.37 | 0.91 |
|  | Smoked | 231 | 1.32 | 0.81 - 2.15 | 0.26 |

Significant results are marked in bold. MUC, mucins; OS, overall survival; LUAD, lung adenocarcinoma; HR, hazard ratio; CI, confidence intervals.

**Supplementary Table 1C**. Correlation between MUC mRNA level and OS in LUSC patients with smoking status.

| MUC family | Smoking status | Cases | HR | 95% CI | P-value |
| --- | --- | --- | --- | --- | --- |
| MUC1 | Smoked | 244 | 0.89 | 0.61 - 1.29 | 0.53 |
| MUC2 | Smoked | 244 | 0.99 | 0.68 - 1.43 | 0.94 |
| MUC3A | Smoked | 244 | 1.2 | 0.83 - 1.74 | 0.34 |
| MUC3B | Smoked | 244 | 1.2 | 0.83 - 1.74 | 0.34 |
| MUC4 | Smoked | 244 | 0.9 | 0.62 - 1.3 | 0.57 |
| MUC5AC | Smoked | 244 | 1.2 | 0.83 - 1.74 | 0.34 |
| MUC5B | Smoked | 244 | 1.08 | 0.75 - 1.57 | 0.67 |
| MUC6 | Smoked | 244 | 1.35 | 0.93 - 1.95 | 0.12 |
| MUC7 | Smoked | 244 | 1.12 | 0.77 - 1.62 | 0.55 |
| MUC8 | Smoked | 244 | 1.48 | 1.02 - 2.15 | **0.037** |
| MUC12 | Smoked | 58 | 1.03 | 0.43 - 2.45 | 0.95 |
| MUC13 | Smoked | 244 | 0.89 | 0.62 - 1.3 | 0.56 |
| MUC15 | Smoked | 58 | 1.11 | 0.45 - 2.72 | 0.83 |
| MUC16 | Smoked | 244 | 1.16 | 0.8 - 1.68 | 0.43 |
| MUC17 | Smoked | 58 | 1.14 | 0.48 - 2.69 | 0.77 |
| MUC19 | Smoked | 58 | 2.26 | 0.91 - 5.64 | 0.073 |
| MUC20 | Smoked | 58 | 1.16 | 0.48 - 2.81 | 0.75 |

Significant results are marked in bold. MUC, mucins; OS, overall survival; LUSC, lung squamous cell carcinoma; HR, hazard ratio; CI, confidence intervals.
