# Supplementary material for: Expression of Mucin Family Proteins in Non-Small-Cell Lung Cancer and its Role in Evaluation of Prognosis
Source: J Oncol. 2022 Aug 26;2022:4181658. doi: 10.1155/2022/4181658 (PMC9439898; doi:10.1155/2022/4181658)
Supplement: Supplementary Materials — Supplementary Figure 1: representive IHC images of MUC protein expression in normal, LUAD, and LUSC tissue in the HPA (MUC20 and MUC22 were not available). IHC: immunohistochemistry; Ab: antibody; MUC: mucins; LUAD: lung adenocarcinoma; LUSC: lung squamous cell carcinoma; HPA: Human Protein Atlas. Supplementary Table 1A: correlation between MUC mRNA levels and OS in patients with NSCLC with smoking statuses. Significant results are marked in bold. MUCs: mucins; OS: overall survival; NSCLC: non-small-cell lung cancer; HR: hazard ratio; CI: confidence intervals. Supplementary Table 1B: correlation between MUC mRNA levels and OS in patients with LUAD with smoking statuses. Significant results are marked in bold. MUCs: mucins; OS: overall survival; LUAD: lung adenocarcinoma; HR: hazard ratio; CI: confidence intervals. Supplementary Table 1C: correlation between MUC mRNA levels and OS in patients with LUSC with smoking statuses. Significant results are marked in bold. MUCs: mucins; OS: overall survival; LUSC: lung squamous cell carcinoma; HR: hazard ratio; CI: confidence intervals. Supplementary Table 2A: correlation between MUC mRNA levels and OS in patients with NSCLC at different clinical stages. Significant results are marked in bold. MUC: mucins; OS: overall survival; NSCLC: non-small-cell lung cancer; HR: hazard ratio; CI: confidence intervals. Supplementary Table 2B: correlation between MUC mRNA levels and OS in patients with LUAD at different clinical stages. Significant results are marked in bold. MUC: mucins; OS: overall survival; LUAD: lung adenocarcinoma; HR: hazard ratio; CI: confidence intervals. Supplementary Table 2C: correlation between MUC mRNA levels and OS in patients with LUSC at different clinical stages. Significant results are marked in bold. MUC: mucins; OS: overall survival; LUSC: lung squamous cell carcinoma; HR: hazard ratio; CI: confidence intervals. Supplementary Table 3: correlation between MUC mRNA levels and OS in patients with NSCLC acco [file 4181658.f1.zip › Supplementary_Table_2.DOC]

**Supplementary Table 2A**. Correlation between MUC mRNA level and OS in NSCLC patients with clinical stages.

| MUC family | Clinical stages | Cases | HR | 95% CI | P-value |
| --- | --- | --- | --- | --- | --- |
| MUC1 | I | 577 | 0.45 | 0.34 - 0.6 | **1.7e-08** |
|  | II | 244 | 0.8 | 0.56 - 1.16 | 0.24 |
|  | III | 70 | 1.12 | 0.65 - 1.93 | 0.68 |
| MUC2 | I | 577 | 1.14 | 0.87 - 1.49 | 0.35 |
|  | II | 244 | 1.3 | 0.9 - 1.88 | 0.16 |
|  | III | 70 | 0.81 | 0.47 - 1.4 | 0.45 |
| MUC3A | I | 577 | 1.15 | 0.87 - 1.5 | 0.32 |
|  | II | 244 | 1.72 | 1.19 - 2.48 | **0.0036** |
|  | III | 70 | 1 | 0.57 - 1.77 | 0.99 |
| MUC3B | I | 577 | 1.15 | 0.87 - 1.5 | 0.32 |
|  | II | 244 | 1.72 | 1.19 - 2.48 | **0.0036** |
|  | III | 70 | 1 | 0.57 - 1.77 | 0.99 |
| MUC4 | I | 577 | 0.89 | 0.68 - 1.17 | 0.4 |
|  | II | 244 | 0.92 | 0.64 - 1.32 | 0.64 |
|  | III | 70 | 0.96 | 0.56 - 1.65 | 0.89 |
| MUC5AC | I | 577 | 0.97 | 0.74 - 1.27 | 0.81 |
|  | II | 244 | 0.97 | 0.67 - 1.41 | 0.89 |
|  | III | 70 | 1.11 | 0.64 - 1.93 | 0.7 |
| MUC5B | I | 577 | 1.06 | 0.81 - 1.39 | 0.65 |
|  | II | 244 | 0.85 | 0.59 - 1.22 | 0.37 |
|  | III | 70 | 1.42 | 0.82 - 2.45 | 0.21 |
| MUC6 | I | 577 | 1.03 | 0.79 - 1.35 | 0.84 |
|  | II | 244 | 1.32 | 0.91 - 1.9 | 0.14 |
|  | III | 70 | 0.61 | 0.35 - 1.06 | 0.078 |
| MUC7 | I | 577 | 0.87 | 0.66 - 1.15 | 0.34 |
|  | II | 244 | 1 | 0.7 - 1.45 | 0.98 |
|  | III | 70 | 0.81 | 0.47 - 1.4 | 0.45 |
| MUC8 | I | 577 | 1.3 | 1 - 1.71 | 0.053 |
|  | II | 244 | 1.64 | 1.14 - 2.37 | **0.0078** |
|  | III | 70 | 1.02 | 0.59 - 1.77 | 0.93 |
| MUC12 | I | 449 | 1.54 | 1.13 - 2.1 | **0.0062** |
|  | II | 244 | 1.17 | 0.74 - 1.84 | 0.5 |
| MUC13 | I | 577 | 1.24 | 0.95 - 1.62 | 0.12 |
|  | II | 244 | 1.28 | 0.89 - 1.84 | 0.19 |
|  | III | 70 | 0.72 | 0.42 - 1.25 | 0.24 |
| MUC15 | I | 449 | 0.5 | 0.36 - 0.69 | **1.7e - 05** |
|  | II | 161 | 0.64 | 0.4 - 1 | 0.05 |
| MUC16 | I | 577 | 1.19 | 0.91 - 1.56 | 0.2 |
|  | II | 244 | 1.25 | 0.87 - 1.81 | 0.23 |
|  | III | 70 | 1.43 | 0.83 - 2.48 | 0.2 |
| MUC17 | I | 449 | 1.25 | 0.92 - 1.71 | 0.15 |
|  | II | 161 | 1.51 | 0.96 - 2.37 | 0.075 |
| MUC19 | I | 449 | 0.7 | 0.51 - 0.96 | **0.028** |
|  | II | 161 | 0.62 | 0.39 - 0.98 | **0.041** |
| MUC20 | I | 449 | 0.76 | 0.56 - 1.04 | 0.081 |
|  | II | 161 | 1.34 | 0.85 - 2.12 | 0.2 |

Significant results are marked in bold. MUC, mucins; OS, overall survival; NSCLC, non-small cell lung cancer; HR, hazard ratio; CI, confidence intervals.

**Supplementary Table 2B**. Correlation between MUC mRNA level and OS in LUAD patients with clinical stages.

| MUC family | Clinical stages | Cases | HR | 95% CI | P-value |
| --- | --- | --- | --- | --- | --- |
| MUC1 | I | 370 | 0.93 | 0.63 - 1.36 | 0.7 |
|  | II | 136 | 0.88 | 0.55 - 1.43 | 0.61 |
| MUC2 | I | 370 | 0.89 | 0.61 - 1.31 | 0.56 |
|  | II | 136 | 1.07 | 0.66 - 1.72 | 0.79 |
| MUC3A | I | 370 | 1.29 | 0.88 - 1.9 | 0.19 |
|  | II | 136 | 1.74 | 1.07 - 2.83 | **0.025** |
| MUC3B | I | 370 | 1.29 | 0.88 - 1.9 | 0.19 |
|  | II | 136 | 1.74 | 1.07 - 2.83 | **0.025** |
| MUC4 | I | 370 | 1.13 | 0.77 - 1.67 | 0.52 |
|  | II | 136 | 1.12 | 0.69 - 1.82 | 0.64 |
| MUC5AC | I | 370 | 1.01 | 0.69 - 1.48 | 0.96 |
|  | II | 136 | 1.01 | 0.63 - 1.64 | 0.95 |
| MUC5B | I | 370 | 1.56 | 1.05 - 2.32 | **0.026** |
|  | II | 136 | 1.04 | 0.64 - 1.7 | 0.86 |
| MUC6 | I | 370 | 1 | 0.68 - 1.47 | 0.99 |
|  | II | 136 | 1.09 | 0.67 - 1.77 | 0.72 |
| MUC7 | I | 370 | 0.77 | 0.52 - 1.15 | 0.2 |
|  | II | 136 | 1.03 | 0.63 - 1.68 | 0.92 |
| MUC8 | I | 370 | 1.04 | 0.71 - 1.53 | 0.83 |
|  | II | 136 | 1.91 | 1.17 - 3.14 | **0.0088** |
| MUC12 | I | 346 | 2.15 | 1.42 - 3.25 | **2e - 04** |
|  | II | 118 | 1.76 | 1.03 - 3 | **0.037** |
| MUC13 | I | 370 | 1.17 | 0.8 - 1.72 | 0.42 |
|  | II | 136 | 1.77 | 1.09 - 2.89 | **0.02** |
| MUC15 | I | 346 | 0.46 | 0.31 - 0.71 | **0.00025** |
|  | II | 118 | 0.64 | 0.38 - 1.07 | 0.088 |
| MUC16 | I | 370 | 1.4 | 0.95 - 2.07 | 0.087 |
|  | II | 136 | 1.19 | 0.74 - 1.93 | 0.48 |
| MUC17 | I | 346 | 1.18 | 0.8 - 1.77 | 0.4 |
|  | II | 118 | 1.46 | 0.87 - 2.47 | 0.15 |
| MUC19 | I | 346 | 0.42 | 0.27 - 0.65 | **6.7e - 05** |
|  | II | 118 | 0.83 | 0.49 - 1.4 | 0.48 |
| MUC20 | I | 346 | 1.15 | 0.77 - 1.71 | 0.5 |
|  | II | 118 | 1.37 | 0.81 - 3.32 | 0.24 |

Significant results are marked in bold. MUC, mucins; OS, overall survival; LUAD, lung adenocarcinoma; HR, hazard ratio; CI, confidence intervals.

**Supplementary Table 2C**. Correlation between MUC mRNA level and OS in LUSC patients with clinical stages.

| MUC family | Clinical stages | Cases | HR | 95% CI | P-value |
| --- | --- | --- | --- | --- | --- |
| MUC1 | I | 172 | 1.02 | 0.67 - 1.56 | 0.93 |
|  | II | 100 | 0.79 | 0.43 - 1.43 | 0.43 |
| MUC2 | I | 172 | 0.71 | 0.46 - 1.1 | 0.12 |
|  | II | 100 | 1 | 0.55 - 1.83 | 0.99 |
| MUC3A | I | 172 | 0.97 | 0.63 - 1.49 | 0.87 |
|  | II | 100 | 2.01 | 1.09 - 3.71 | **0.022** |
| MUC3B | I | 172 | 0.97 | 0.63 - 1.49 | 0.87 |
|  | II | 100 | 2.01 | 1.09 - 3.71 | **0.022** |
| MUC4 | I | 172 | 0.64 | 0.42 - 0.98 | **0.04** |
|  | II | 100 | 0.45 | 0.24 - 0.85 | **0.011** |
| MUC5AC | I | 172 | 1.01 | 0.66 - 1.55 | 0.97 |
|  | II | 100 | 0.67 | 0.36 - 1.25 | 0.2 |
| MUC5B | I | 172 | 1.27 | 0.83 - 1.95 | 0.27 |
|  | II | 100 | 1.45 | 0.79 - 2.66 | 0.23 |
| MUC6 | I | 172 | 1.13 | 0.74 - 1.74 | 0.56 |
|  | II | 100 | 1.52 | 0.83 - 2.78 | 0.18 |
| MUC7 | I | 172 | 0.9 | 0.58 - 1.39 | 0.64 |
|  | II | 100 | 1.03 | 0.56 - 1.88 | 0.93 |
| MUC8 | I | 172 | 1.25 | 0.81 - 1.93 | 0.31 |
|  | II | 100 | 1.42 | 0.78 - 2.61 | 0.25 |
| MUC12 | I | 74 | 0.7 | 0.38 - 1.29 | 0.25 |
| MUC13 | I | 172 | 0.82 | 0.53 - 1.26 | 0.36 |
|  | II | 100 | 0.73 | 0.4 - 1.33 | 0.3 |
|  | III | 70 | 0.72 | 0.42 - 1.25 | 0.24 |
| MUC15 | I | 74 | 0.95 | 0.53 - 1.73 | 0.87 |
| MUC16 | I | 172 | 1.21 | 0.79 - 1.85 | 0.38 |
|  | II | 100 | 0.86 | 0.47 - 1.57 | 0.62 |
| MUC17 | I | 74 | 1.21 | 0.67 - 2.2 | 0.52 |
| MUC19 | I | 74 | 2.17 | 1.18 - 4 | **0.011** |
| MUC20 | I | 74 | 1 | 0.55 - 1.81 | 1 |

Significant results are marked in bold. MUC, mucins; OS, overall survival; LUSC, lung squamous cell carcinoma; HR, hazard ratio; CI, confidence intervals.
