# Supplementary material for: Expression of Mucin Family Proteins in Non-Small-Cell Lung Cancer and its Role in Evaluation of Prognosis
Source: J Oncol. 2022 Aug 26;2022:4181658. doi: 10.1155/2022/4181658 (PMC9439898; doi:10.1155/2022/4181658)
Supplement: Supplementary Materials — Supplementary Figure 1: representive IHC images of MUC protein expression in normal, LUAD, and LUSC tissue in the HPA (MUC20 and MUC22 were not available). IHC: immunohistochemistry; Ab: antibody; MUC: mucins; LUAD: lung adenocarcinoma; LUSC: lung squamous cell carcinoma; HPA: Human Protein Atlas. Supplementary Table 1A: correlation between MUC mRNA levels and OS in patients with NSCLC with smoking statuses. Significant results are marked in bold. MUCs: mucins; OS: overall survival; NSCLC: non-small-cell lung cancer; HR: hazard ratio; CI: confidence intervals. Supplementary Table 1B: correlation between MUC mRNA levels and OS in patients with LUAD with smoking statuses. Significant results are marked in bold. MUCs: mucins; OS: overall survival; LUAD: lung adenocarcinoma; HR: hazard ratio; CI: confidence intervals. Supplementary Table 1C: correlation between MUC mRNA levels and OS in patients with LUSC with smoking statuses. Significant results are marked in bold. MUCs: mucins; OS: overall survival; LUSC: lung squamous cell carcinoma; HR: hazard ratio; CI: confidence intervals. Supplementary Table 2A: correlation between MUC mRNA levels and OS in patients with NSCLC at different clinical stages. Significant results are marked in bold. MUC: mucins; OS: overall survival; NSCLC: non-small-cell lung cancer; HR: hazard ratio; CI: confidence intervals. Supplementary Table 2B: correlation between MUC mRNA levels and OS in patients with LUAD at different clinical stages. Significant results are marked in bold. MUC: mucins; OS: overall survival; LUAD: lung adenocarcinoma; HR: hazard ratio; CI: confidence intervals. Supplementary Table 2C: correlation between MUC mRNA levels and OS in patients with LUSC at different clinical stages. Significant results are marked in bold. MUC: mucins; OS: overall survival; LUSC: lung squamous cell carcinoma; HR: hazard ratio; CI: confidence intervals. Supplementary Table 3: correlation between MUC mRNA levels and OS in patients with NSCLC acco [file 4181658.f1.zip › Supplementary_Table_3 (1).DOC]

**Supplementary Table 3**. Correlation between MUC mRNA level and OS in NSCLC patients with chemotherapy status.

| MUC family | Chemotherapy | Cases | HR | 95% CI | P-value |
| --- | --- | --- | --- | --- | --- |
| MUC1 | No | 310 | 0.69 | 0.49 - 0.97 | **0.03** |
|  | Yes | 176 | 0.78 | 0.52 - 1.17 | 0.23 |
| MUC2 | No | 310 | 0.88 | 0.63 - 1.23 | 0.44 |
|  | Yes | 176 | 0.81 | 0.54 - 1.22 | 0.31 |
| MUC3A | No | 310 | 0.67 | 0.48 - 0.94 | **0.021** |
|  | Yes | 176 | 0.69 | 0.46 - 1.03 | 0.069 |
| MUC3B | No | 310 | 0.67 | 0.48 - 0.94 | **0.021** |
|  | Yes | 176 | 0.69 | 0.46 - 1.03 | 0.069 |
| MUC4 | No | 310 | 0.81 | 0.58 - 1.13 | 0.22 |
|  | Yes | 176 | 0.93 | 0.62 - 1.39 | 0.72 |
| MUC5AC | No | 310 | 1.26 | 0.9 - 1.76 | 0.18 |
|  | Yes | 176 | 1.13 | 0.75 - 1.7 | 0.55 |
| MUC5B | No | 310 | 1.12 | 0.8 - 1.56 | 0.51 |
|  | Yes | 176 | 1.01 | 0.68 - 1.52 | 0.95 |
| MUC6 | No | 310 | 1.04 | 0.75 - 1.45 | 0.81 |
|  | Yes | 176 | 0.76 | 0.51 - 1.15 | 0.2 |
| MUC7 | No | 310 | 1.16 | 0.83 - 1.62 | 0.38 |
|  | Yes | 176 | 1.11 | 0.73 - 1.68 | 0.63 |
| MUC8 | No | 310 | 1.23 | 0.88 - 1.72 | 0.23 |
|  | Yes | 176 | 0.95 | 0.63 - 1.43 | 0.82 |
| MUC12 | No | 310 | 2.24 | 0.41 - 12.28 | 0.34 |
|  | Yes | 176 | 0.58 | 0.17 - 1.94 | 0.37 |
| MUC13 | No | 310 | 0.93 | 0.67 - 1.3 | 0.68 |
|  | Yes | 176 | 0.81 | 0.54 - 1.22 | 0.32 |
| MUC16 | No | 310 | 1.5 | 1.07 - 2.11 | **0.018** |
|  | Yes | 176 | 1.12 | 0.74 - 1.67 | 0.59 |

Significant results are marked in bold. MUC, mucins; OS, overall survival; NSCLC, non-small cell lung cancer; HR, hazard ratio; CI, confidence intervals.
