# Supplementary material for: Expression of Mucin Family Proteins in Non-Small-Cell Lung Cancer and its Role in Evaluation of Prognosis
Source: J Oncol. 2022 Aug 26;2022:4181658. doi: 10.1155/2022/4181658 (PMC9439898; doi:10.1155/2022/4181658)
Supplement: Supplementary Materials — Supplementary Figure 1: representive IHC images of MUC protein expression in normal, LUAD, and LUSC tissue in the HPA (MUC20 and MUC22 were not available). IHC: immunohistochemistry; Ab: antibody; MUC: mucins; LUAD: lung adenocarcinoma; LUSC: lung squamous cell carcinoma; HPA: Human Protein Atlas. Supplementary Table 1A: correlation between MUC mRNA levels and OS in patients with NSCLC with smoking statuses. Significant results are marked in bold. MUCs: mucins; OS: overall survival; NSCLC: non-small-cell lung cancer; HR: hazard ratio; CI: confidence intervals. Supplementary Table 1B: correlation between MUC mRNA levels and OS in patients with LUAD with smoking statuses. Significant results are marked in bold. MUCs: mucins; OS: overall survival; LUAD: lung adenocarcinoma; HR: hazard ratio; CI: confidence intervals. Supplementary Table 1C: correlation between MUC mRNA levels and OS in patients with LUSC with smoking statuses. Significant results are marked in bold. MUCs: mucins; OS: overall survival; LUSC: lung squamous cell carcinoma; HR: hazard ratio; CI: confidence intervals. Supplementary Table 2A: correlation between MUC mRNA levels and OS in patients with NSCLC at different clinical stages. Significant results are marked in bold. MUC: mucins; OS: overall survival; NSCLC: non-small-cell lung cancer; HR: hazard ratio; CI: confidence intervals. Supplementary Table 2B: correlation between MUC mRNA levels and OS in patients with LUAD at different clinical stages. Significant results are marked in bold. MUC: mucins; OS: overall survival; LUAD: lung adenocarcinoma; HR: hazard ratio; CI: confidence intervals. Supplementary Table 2C: correlation between MUC mRNA levels and OS in patients with LUSC at different clinical stages. Significant results are marked in bold. MUC: mucins; OS: overall survival; LUSC: lung squamous cell carcinoma; HR: hazard ratio; CI: confidence intervals. Supplementary Table 3: correlation between MUC mRNA levels and OS in patients with NSCLC acco [file 4181658.f1.zip › Supplementary_Table_4 (1).DOC]

**Supplementary Table 4A**. Correlation between MUC mRNA level and OS in NSCLC patients with different sex.

| MUC family | Sex | Cases | HR | 95% CI | P-value |
| --- | --- | --- | --- | --- | --- |
| MUC1 | Female | 714 | 0.81 | 0.64 - 1.02 | 0.069 |
|  | Male | 1,100 | 0.69 | 0.59 - 0.81 | **5.1e-06** |
| MUC2 | Female | 714 | 1.21 | 0.96 - 1.52 | 0.11 |
|  | Male | 1,100 | 1.15 | 0.98 - 1.35 | **0.076** |
| MUC3A | Female | 714 | 1.43 | 1.13 - 1.8 | **0.0027** |
|  | Male | 1,100 | 1.04 | 0.89 - 1.22 | 0.63 |
| MUC3B | Female | 714 | 1.43 | 1.13 - 1.8 | **0.0027** |
|  | Male | 1,100 | 1.04 | 0.89 - 1.22 | 0.63 |
| MUC4 | Female | 714 | 1 | 0.79 - 1.26 | 0.99 |
|  | Male | 1,100 | 0.97 | 0.83 - 1.13 | 0.7 |
| MUC5AC | Female | 714 | 1.03 | 0.82 - 1.3 | 0.78 |
|  | Male | 1,100 | 1.11 | 0.95 - 1.3 | 0.2 |
| MUC5B | Female | 714 | 0.97 | 0.77 - 1.23 | 0.83 |
|  | Male | 1,100 | 0.96 | 0.82 - 1.13 | 0.63 |
| MUC6 | Female | 714 | 1.26 | 1 - 1.59 | **0.047** |
|  | Male | 1,100 | 1.02 | 0.87 - 1.19 | 0.84 |
| MUC7 | Female | 714 | 1.05 | 0.83 - 1.32 | 0.68 |
|  | Male | 1,100 | 0.95 | 0.81 - 1.12 | 0.56 |
| MUC8 | Female | 714 | 1.46 | 1.15 - 1.84 | **0.0015** |
|  | Male | 1,100 | 0.97 | 0.83 - 1.13 | 0.68 |
| MUC12 | Female | 714 | 1.66 | 1.18 - 2.33 | **0.0031** |
|  | Male | 1,100 | 1.14 | 0.93 - 1.4 | 0.2 |
| MUC13 | Female | 714 | 1.18 | 0.94 - 1.49 | 0.16 |
|  | Male | 1,100 | 1 | 0.86 - 1.17 | 0.99 |
| MUC15 | Female | 714 | 0.76 | 0.54 - 1.06 | 0.1 |
|  | Male | 1,100 | 0.68 | 0.56 - 0.84 | **0.00023** |
| MUC16 | Female | 714 | 1.29 | 1.02 - 1.63 | **0.032** |
|  | Male | 1,100 | 1.23 | 1.05 - 1.44 | **0.009** |
| MUC17 | Female | 374 | 1.38 | 0.99 - 1.93 | 0.06 |
|  | Male | 659 | 1.14 | 0.93 - 1.39 | 0.21 |
| MUC19 | Female | 714 | 0.81 | 0.58 - 1.13 | 0.22 |
|  | Male | 1,100 | 1.03 | 0.84 - 1.26 | 0.8 |
| MUC20 | Female | 374 | 0.81 | 0.58 - 1.13 | 0.22 |
|  | Male | 659 | 0.92 | 0.75 - 1.13 | 0.42 |

Significant results are marked in bold. MUC, mucins; OS, overall survival; NSCLC, non-small cell lung cancer; HR, hazard ratio; CI, confidence intervals.

**Supplementary Table 4B**. Correlation between MUC mRNA level and OS in LUAD patients with different sex.

| MUC family | Sex | Cases | HR | 95% CI | P-value |
| --- | --- | --- | --- | --- | --- |
| MUC1 | Female | 317 | 0.75 | 0.51 - 1.09 | 0.13 |
|  | Male | 344 | 0.67 | 0.48 - 0.94 | **0.019** |
| MUC2 | Female | 317 | 0.95 | 0.65 - 1.39 | 0.8 |
|  | Male | 344 | 0.99 | 0.71 - 1.37 | 0.95 |
| MUC3A | Female | 317 | 1.49 | 1.02 - 2.17 | **0.04** |
|  | Male | 344 | 1.21 | 0.87 - 1.67 | 0.25 |
| MUC3B | Female | 317 | 1.49 | 1.02 - 2.17 | **0.04** |
|  | Male | 344 | 1.21 | 0.87 - 1.67 | 0.25 |
| MUC4 | Female | 317 | 1.31 | 0.89 - 1.91 | 0.17 |
|  | Male | 344 | 1.2 | 0.86 - 1.66 | 0.28 |
| MUC5AC | Female | 317 | 0.84 | 0.57 - 1.23 | 0.36 |
|  | Male | 344 | 1.22 | 0.88 - 1.69 | 0.23 |
| MUC5B | Female | 317 | 1.16 | 0.79 - 1.69 | 0.45 |
|  | Male | 344 | 1.41 | 1.01 - 1.96 | **0.043** |
| MUC6 | Female | 317 | 1.24 | 0.85 - 1.82 | 0.26 |
|  | Male | 344 | 0.85 | 0.61 - 1.18 | 0.34 |
| MUC7 | Female | 317 | 0.84 | 0.57 - 1.23 | 0.37 |
|  | Male | 344 | 0.87 | 0.63 - 1.22 | 0.42 |
| MUC8 | Female | 317 | 1.75 | 1.2 - 2.56 | **0.0035** |
|  | Male | 344 | 1.1 | 0.79 - 1.52 | 0.57 |
| MUC12 | Female | 286 | 1.72 | 1.14 - 2.59 | **0.0083** |
|  | Male | 328 | 1.47 | 1.05 - 2.06 | **0.024** |
| MUC13 | Female | 317 | 1.36 | 0.93 - 1.99 | 0.11 |
|  | Male | 344 | 1.04 | 0.75 - 1.45 | 0.79 |
| MUC15 | Female | 286 | 0.6 | 0.4 - 0.9 | **0.014** |
|  | Male | 328 | 0.62 | 0.44 - 0.87 | **0.0054** |
| MUC16 | Female | 317 | 1.3 | 0.89 - 1.9 | 0.18 |
|  | Male | 344 | 2.03 | 1.45 - 2.85 | **2.5e - 05** |
| MUC17 | Female | 286 | 1.19 | 0.8 - 1.78 | 0.39 |
|  | Male | 328 | 1.22 | 0.87 - 1.7 | 0.25 |
| MUC19 | Female | 286 | 0.69 | 0.46 - 1.04 | 0.074 |
|  | Male | 328 | 0.86 | 0.61 - 1.2 | 0.37 |
| MUC20 | Female | 286 | 0.85 | 0.57 - 1.27 | 0.43 |
|  | Male | 328 | 1.18 | 0.84 - 1.64 | 0.34 |

Significant results are marked in bold. MUC, mucins; OS, overall survival; LUAD, lung adenocarcinoma; HR, hazard ratio; CI, confidence intervals.

**Supplementary Table 4C**. Correlation between MUC mRNA level and OS in LUSC patients with different sex.

| MUC family | Sex | Cases | HR | 95% CI | P-value |
| --- | --- | --- | --- | --- | --- |
| MUC1 | Female | 129 | 0.86 | 0.49 - 1.5 | 0.59 |
|  | Male | 342 | 1.01 | 0.77 - 1.34 | 0.93 |
| MUC2 | Female | 129 | 0.79 | 0.45 - 1.39 | 0.42 |
|  | Male | 342 | 1.15 | 0.87 - 1.53 | 0.32 |
| MUC3A | Female | 129 | 1.46 | 0.83 - 2.58 | 0.19 |
|  | Male | 342 | 1.31 | 0.99 - 1.73 | 0.057 |
| MUC3B | Female | 129 | 1.46 | 0.83 - 2.58 | 0.19 |
|  | Male | 342 | 1.31 | 0.99 - 1.73 | 0.057 |
| MUC4 | Female | 129 | 0.55 | 0.31 - 0.98 | **0.039** |
|  | Male | 342 | 1 | 0.76 - 1.32 | 0.98 |
| MUC5AC | Female | 129 | 0.94 | 0.54 - 1.65 | 0.84 |
|  | Male | 342 | 0.93 | 0.7 - 1.23 | 0.61 |
| MUC5B | Female | 129 | 1.06 | 0.61 - 1.84 | 0.85 |
|  | Male | 342 | 1.17 | 0.88 - 1.54 | 0.27 |
| MUC6 | Female | 129 | 1.55 | 0.88 - 2.74 | 0.12 |
|  | Male | 342 | 1.18 | 0.9 - 1.56 | 0.24 |
| MUC7 | Female | 129 | 0.61 | 0.35 - 1.08 | 0.086 |
|  | Male | 342 | 1 | 0.76 - 1.33 | 0.99 |
| MUC8 | Female | 129 | 1.28 | 0.73 - 2.24 | 0.39 |
|  | Male | 342 | 1 | 0.76 - 1.33 | 0.99 |
| MUC12 | Female | 129 | 1.14 | 0.65 - 1.99 | 0.65 |
|  | Male | 342 | 1.05 | 0.79 - 1.38 | 0.76 |
| MUC13 | Female | 129 | 0.98 | 0.56 - 1.71 | 0.93 |
|  | Male | 342 | 1.09 | 0.83 - 1.45 | 0.53 |
| MUC15 | Female | 51 | 1.74 | 0.74 - 4.06 | 0.2 |
|  | Male | 167 | 0.74 | 0.51 - 1.07 | 0.11 |
| MUC16 | Female | 129 | 1.14 | 0.65 - 1.99 | 0.65 |
|  | Male | 342 | 1.05 | 0.79 - 1.38 | 0.76 |
| MUC17 | Female | 51 | 1.53 | 0.67 - 3.49 | 0.31 |
|  | Male | 167 | 1.01 | 0.7 - 1.47 | 0.95 |
| MUC19 | Female | 51 | 2.19 | 2.19 - 5.21 | 0.069 |
|  | Male | 167 | 1.23 | 0.85 - 1.79 | 0.27 |
| MUC20 | Female |  |  |  |  |
|  | Male |  |  |  |  |

Significant results are marked in bold. MUC, mucins; OS, overall survival; LUSC, lung squamous cell carcinoma; HR, hazard ratio; CI, confidence intervals.
