# Supplementary material for: Expression of Mucin Family Proteins in Non-Small-Cell Lung Cancer and its Role in Evaluation of Prognosis
Source: J Oncol. 2022 Aug 26;2022:4181658. doi: 10.1155/2022/4181658 (PMC9439898; doi:10.1155/2022/4181658)
Supplement: Supplementary Materials — Supplementary Figure 1: representive IHC images of MUC protein expression in normal, LUAD, and LUSC tissue in the HPA (MUC20 and MUC22 were not available). IHC: immunohistochemistry; Ab: antibody; MUC: mucins; LUAD: lung adenocarcinoma; LUSC: lung squamous cell carcinoma; HPA: Human Protein Atlas. Supplementary Table 1A: correlation between MUC mRNA levels and OS in patients with NSCLC with smoking statuses. Significant results are marked in bold. MUCs: mucins; OS: overall survival; NSCLC: non-small-cell lung cancer; HR: hazard ratio; CI: confidence intervals. Supplementary Table 1B: correlation between MUC mRNA levels and OS in patients with LUAD with smoking statuses. Significant results are marked in bold. MUCs: mucins; OS: overall survival; LUAD: lung adenocarcinoma; HR: hazard ratio; CI: confidence intervals. Supplementary Table 1C: correlation between MUC mRNA levels and OS in patients with LUSC with smoking statuses. Significant results are marked in bold. MUCs: mucins; OS: overall survival; LUSC: lung squamous cell carcinoma; HR: hazard ratio; CI: confidence intervals. Supplementary Table 2A: correlation between MUC mRNA levels and OS in patients with NSCLC at different clinical stages. Significant results are marked in bold. MUC: mucins; OS: overall survival; NSCLC: non-small-cell lung cancer; HR: hazard ratio; CI: confidence intervals. Supplementary Table 2B: correlation between MUC mRNA levels and OS in patients with LUAD at different clinical stages. Significant results are marked in bold. MUC: mucins; OS: overall survival; LUAD: lung adenocarcinoma; HR: hazard ratio; CI: confidence intervals. Supplementary Table 2C: correlation between MUC mRNA levels and OS in patients with LUSC at different clinical stages. Significant results are marked in bold. MUC: mucins; OS: overall survival; LUSC: lung squamous cell carcinoma; HR: hazard ratio; CI: confidence intervals. Supplementary Table 3: correlation between MUC mRNA levels and OS in patients with NSCLC acco [file 4181658.f1.zip › Supplementary_Table_5 (1).DOC]

**Supplementary Table 5**. The information of 13 NSCLC datasets.

| No. | Studies | Samples | Pubmed |
| --- | --- | --- | --- |
| 1 | Lung Adenocarcinoma (Broad, Cell 2012) | 75 | https://www.ncbi.nlm.nih.gov/pubmed/22980975 |
| 2 | Lung Adenocarcinoma (MSKCC, Science 2015) | 35 | https://www.ncbi.nlm.nih.gov/pubmed/25765070 |
| 3 | Lung Adenocarcinoma (OncoSG, Nat Genet 2020) | 305 | https://www.ncbi.nlm.nih.gov/pubmed/32015526 |
| 4 | Lung Adenocarcinoma (TCGA, Firehose Legacy) | 586 |  |
| 5 | Lung Adenocarcinoma (TSP, Nature 2008) | 163 | https://www.ncbi.nlm.nih.gov/pubmed/18948947 |
| 6 | Lung Squamous Cell Carcinoma (TCGA, Firehose Legacy) | 511 |  |
| 7 | Non-Small Cell Cancer (MSKCC, Cancer Discov 2017) | 915 | https://www.ncbi.nlm.nih.gov/pubmed/28336552 |
| 8 | Non-Small Cell Cancer (MSK, Cancer Cell 2018) | 75 | https://www.ncbi.nlm.nih.gov/pubmed/29657128 |
| 9 | Non-Small Cell Cancer (MSK, Science 2015) | 16 | https://www.ncbi.nlm.nih.gov/pubmed/25765070 |
| 10 | Non-Small Cell Cancer (MSKCC, J Clin Oncol 2018) | 240 | https://www.ncbi.nlm.nih.gov/pubmed/29337640 |
| 11 | Non-Small Cell Cancer (TRACERx, NEJM & Nature 2017) | 447 | https://www.ncbi.nlm.nih.gov/pubmed/28445112,28445469 |
| 12 | Pan-Lung Cancer (TCGA, Nat Genet 2016) | 1144 | https://www.ncbi.nlm.nih.gov/pubmed/27158780 |
| 13 | Non-Small Cell Cancer (University of Turin, Lung Cancer 2017) | 41 | https://www.ncbi.nlm.nih.gov/pubmed/27346245 |
