# Supplementary material for: Expression of Mucin Family Proteins in Non-Small-Cell Lung Cancer and its Role in Evaluation of Prognosis
Source: J Oncol. 2022 Aug 26;2022:4181658. doi: 10.1155/2022/4181658 (PMC9439898; doi:10.1155/2022/4181658)
Supplement: Supplementary Materials — Supplementary Figure 1: representive IHC images of MUC protein expression in normal, LUAD, and LUSC tissue in the HPA (MUC20 and MUC22 were not available). IHC: immunohistochemistry; Ab: antibody; MUC: mucins; LUAD: lung adenocarcinoma; LUSC: lung squamous cell carcinoma; HPA: Human Protein Atlas. Supplementary Table 1A: correlation between MUC mRNA levels and OS in patients with NSCLC with smoking statuses. Significant results are marked in bold. MUCs: mucins; OS: overall survival; NSCLC: non-small-cell lung cancer; HR: hazard ratio; CI: confidence intervals. Supplementary Table 1B: correlation between MUC mRNA levels and OS in patients with LUAD with smoking statuses. Significant results are marked in bold. MUCs: mucins; OS: overall survival; LUAD: lung adenocarcinoma; HR: hazard ratio; CI: confidence intervals. Supplementary Table 1C: correlation between MUC mRNA levels and OS in patients with LUSC with smoking statuses. Significant results are marked in bold. MUCs: mucins; OS: overall survival; LUSC: lung squamous cell carcinoma; HR: hazard ratio; CI: confidence intervals. Supplementary Table 2A: correlation between MUC mRNA levels and OS in patients with NSCLC at different clinical stages. Significant results are marked in bold. MUC: mucins; OS: overall survival; NSCLC: non-small-cell lung cancer; HR: hazard ratio; CI: confidence intervals. Supplementary Table 2B: correlation between MUC mRNA levels and OS in patients with LUAD at different clinical stages. Significant results are marked in bold. MUC: mucins; OS: overall survival; LUAD: lung adenocarcinoma; HR: hazard ratio; CI: confidence intervals. Supplementary Table 2C: correlation between MUC mRNA levels and OS in patients with LUSC at different clinical stages. Significant results are marked in bold. MUC: mucins; OS: overall survival; LUSC: lung squamous cell carcinoma; HR: hazard ratio; CI: confidence intervals. Supplementary Table 3: correlation between MUC mRNA levels and OS in patients with NSCLC acco [file 4181658.f1.zip › Supplementary_Table_6 (1).DOC]

**Supplementary Table 6**. The expression and prognostic value of MUC in NSCLC.

| MUC | mRNA level | | Prognostic with unfavourable OS | | Clinicopathological features | | | | | | | |
| --- | --- | --- | --- | --- | --- | --- | --- | --- | --- | --- | --- | --- |
|  |  |  |  |  | Smoked | | Clinical stages | | No chemotherapy | Sex | | |
|  |  |  |  |  |  |  |  |  |  | Female | | Male |
|  | LUAD | LUSC | LUAD | LUSC | LUAD | LUSC | LUAD | LUSC | NSCLC | LUAD | LUSC | LUAD |
| MUC1 |  | ↓ | ▽ | # |  |  |  |  | ※ |  |  | ※ |
| MUC2 |  |  | # |  |  |  |  |  |  |  |  |  |
| MUC3A | ↑ |  | △ |  |  |  | □ | □ | ※ | □ |  |  |
| MUC3B |  |  |  |  |  |  | □ | □ | ※ | □ |  |  |
| MUC4 | ↑ |  |  |  |  |  |  |  |  |  | ※ |  |
| MUC5B | ↑ |  | # |  |  |  | □ |  |  |  |  | □ |
| MUC8 |  |  | △ |  |  | □ | □ | ※ |  | □ |  | □ |
| MUC12 |  |  | △ | # | □ |  | □ |  |  | □ | □ | □ |
| MUC13 | ↑ |  | △ |  |  |  | □ |  |  |  |  |  |
| MUC15 |  |  | ▽ |  | ※ |  | ※ |  |  | ※ |  | ※ |
| MUC16 | ↑ |  | △ |  | □ |  |  |  | □ |  |  | □ |
| MUC17 |  |  | △ |  |  |  |  |  |  |  |  |  |
| MUC19 |  |  |  | △ |  |  | ※ | □ |  |  |  |  |
| MUC20 | ↑ | ↑ |  |  |  |  |  |  |  |  |  |  |
| MUC21 | ↑ |  |  |  |  |  |  |  |  |  |  |  |

MUC, mucins; NSCLC, non-small cell lung cancer; OS, overall survival; LUAD, lung adenocarcinoma; LUSC, lung squamous cell carcinoma; ↓, MUC mRNA low expression in GEPIA; ↑, MUC mRNA high expression in GEPIA; ▽, increased MUC mRNA level was associated with favourable OS in KM Plotter; △, increased MUC mRNA level was associated with unfavourable OS in KM Plotter; #, Increased MUC mRNA expression was correlated with unfavourable OS in GEPIA; □, high MUC mRNA level was correlated with unfavourable OS with the different clinicopathological features in KM Plotter; ※, high MUC mRNA level was correlated with favourable OS with the clinicopathological features in KM Plotter
